# Zika beyond the Americas: Travelers as sentinels of Zika virus transmission. A GeoSentinel analysis, 2012 to 2016.

**DOI:** 10.1371/journal.pone.0185689

**Published:** 2017-10-03

**Authors:** Karin Leder, Martin P. Grobusch, Philippe Gautret, Lin H. Chen, Susan Kuhn, Poh Lian Lim, Johnnie Yates, Anne E. McCarthy, Camilla Rothe, Yasuyuki Kato, Emmanuel Bottieau, Kristina Huber, Eli Schwartz, William Stauffer, Denis Malvy, Marc T. M. Shaw, Christophe Rapp, Lucille Blumberg, Mogens Jensenius, Perry J. J. van Genderen, Davidson H. Hamer, for the GeoSentinel Surveillance Network

**Affiliations:** 1 School of Epidemiology and Preventive Medicine, Monash University, Melbourne, Victoria, Australia; 2 Victorian Infectious Disease Service, Royal Melbourne Hospital at the Doherty Institute for Infection and Immunity, Melbourne, Victoria, Australia; 3 Center of Tropical Medicine and Travel Medicine, Department of Infectious Diseases, Division of Internal Medicine, Academic Medical Center, University of Amsterdam, Amsterdam, The Netherlands; 4 Aix Marseille Université, UM63, CNRS 7278, IRD 198, INSERM 1095, IHU Méditerranée Infection, Marseillle, France; 5 Travel Medicine Center, Mount Auburn Hospital, Cambridge, Massachusetts, United States of America; 6 Harvard Medical School, Boston, Massachusetts, United States of America; 7 University of Calgary, Calgary, Canada; 8 Institute of Infectious Disease & Epidemiology, Tan Tock Seng Hospital, Singapore, Singapore; 9 Lee Kong Chian School of Medicine, Nanyang Technological University, Singapore, Singapore; 10 Hawaii Permenente Medical Group, Honolulu, Hawaii, United States of America; 11 Department of Medicine, The Ottawa Hospital, Ottawa, Canada; 12 University of Ottawa, Ottawa, Canada; 13 University of Hamburg, Division of Tropical Medicine and Infectious Diseases, Hamburg, Germany; 14 Division of Preparedness and Emerging Infections, Disease Control and Prevention Center, National Center for Global Health and Medicine, Tokyo, Japan; 15 Department of Clinical Sciences, Institute of Tropical Medicine, Antwerp, Belgium; 16 Division of Infectious Diseases and Tropical Medicine, Medical Center of the University of Munich (LMU), Munich, Germany; 17 Center for Geographic Medicine and Tropical Diseases, Chaim Sheba Medical Center, Tel Hashomer, Tel Aviv, Israel; 18 Sackler School of Medicine, Tel Aviv University, Tel Aviv, Israel; 19 Department of Medicine and Pediatrics, Infectious Diseases and Internal Medicine, University of Minnesota, Minnesota, United States of America; 20 University Hospital Center & Inserm 1219, University of Bordeaux, Bordeaux, France; 21 James Cook University, Queensland, Australia; 22 CMETE Travel Clinic Paris, Department of Infectious and Tropical Diseases, Begin Military Hospital, Saint-Mandé, France; 23 National Institute for Communicable Diseases, Johannesburg, South Africa; 24 Department of Infectious Diseases, Oslo University Hospital, Oslo, Norway; 25 Institute for Tropical Diseases, Harbour Hospital, Rotterdam, The Netherlands; 26 Department of Global Health and Center for Global Health and Development, Boston University School of Public Health, Boston, Massachusetts, United States of America; 27 Section of Infectious Diseases, Department of Medicine, Boston University School of Medicine, Boston, Massachusetts, United States of America; Institut Pasteur of Shanghai Chinese Academy of Sciences, CHINA

## Abstract

**Background:**

Zika virus (ZIKV) was first isolated in Africa; decades later, caused large outbreaks in the Pacific, and is considered endemic in Asia. We aim to describe ZIKV disease epidemiology outside the Americas, the importance of travelers as sentinels of disease transmission, and discrepancies in travel advisories from major international health organizations.

**Methods and findings:**

This descriptive analysis using GeoSentinel Surveillance Network records involves sixty-four travel and tropical medicine clinics in 29 countries. Ill returned travelers with a confirmed or probable diagnosis of ZIKV disease acquired in Africa, Asia and the Pacific seen between 1 January 2012 and 31 December 2016 are included, and the frequencies of demographic, trip, and diagnostic characteristics described. ZIKV was acquired in Asia (18), the Pacific (10) and Africa (1). For five countries (Indonesia, Philippines, Thailand, Vietnam, Cameroon), GeoSentinel patients were sentinel markers of recent Zika activity. Additionally, the first confirmed ZIKV infection acquired in Kiribati was reported to GeoSentinel (2015), and a probable case was reported from Timor Leste (April 2016), representing the only case known to date. Review of Zika situation updates from major international health authorities for country risk classifications shows heterogeneity in ZIKV country travel advisories.

**Conclusions:**

Travelers are integral to the global spread of ZIKV, serving as sentinel markers of disease activity. Although GeoSentinel data are collected by specialized clinics and do not capture all imported cases, we show that surveillance of imported infections by returned travelers augments local surveillance system data regarding ZIKV epidemiology and can assist with risk categorization by international authorities. However, travel advisories are variable due to risk uncertainties.

## Introduction

Zika virus (ZIKV) made global headlines in 2015 when Brazil identified the first cases of confirmed transmission. The alarming association with congenital abnormalities triggered the World Health Organization (WHO) to declare a public health emergency of international concern on 1 February 2016 [1]. Whereas significant attention has focused on the Americas and travel-associated cases exported from the Americas, including exportation of cases reported to GeoSentinel [2], ZIKV was first isolated from a rhesus macaque in 1947 in the Zika Forest in Uganda [3, 4] and was responsible for large outbreaks in 2007 in the Pacific Islands of Yap (Federated States of Micronesia) and in 2013 in French Polynesia [5, 6]. Based on serological studies, ZIKV has been endemic in certain regions of Africa and of Asia for over 50 years [3, 7–15] and has been silently circulating in West Africa for over 20 years [16], although limitations in serological testing and potential cross-reactivity with other flaviviruses hinder reliable interpretation.

ZIKV is now widely distributed throughout the tropics and sub-tropics, with 75 countries and territories reporting evidence of vector-borne ZIKV transmission between 2015 and 2016 including: three in West and Central Africa (Cape Verde, Guinea-Bissau, Gabon), 13 in the Pacific (American Samoa, Fiji, Marshall Islands, Micronesia, Palau, Samoa, Tonga, New Caledonia, Cook Islands, French Polynesia, Papua New Guinea, Solomon Islands, Vanuatu), and 10 in Asia (Indonesia, Maldives, Thailand, Bangladesh, Singapore, Philippines, Vietnam, Cambodia, Malaysia, Lao People’s Democratic Republic) [17]. In Africa and Asia-Pacific regions, an estimated 2·6 billion people live in areas with competent mosquito vectors and climatic conditions are suitable for local transmission of ZIKV [18].

Despite recent advances in knowledge and research regarding ZIKV, a general lack of disease awareness prior to the current outbreak, misdiagnoses due to the clinical similarity of ZIKV infections with other infectious diseases, and limited access to diagnostic testing have resulted in many unresolved questions regarding the distribution and transmission risks outside the Americas. There is also uncertainty regarding the impact of different strains and the potential for obstetric and neurological complications, particularly in Asia and Africa. To date, all reported Pacific and American Zika isolates and associated neurologic or microcephaly complications have been attributed to the expansion of the Asian lineage [19–21], but cases of microcephaly have been investigated in Guinea-Bissau and Angola, where the current outbreak is caused by the African lineage [17]. While it is thought that Asian and African ZIKV strains differ in their abilities to infect cells of the central nervous system and to cause neurodevelopmental problems [22], residual uncertainties hinder accurate risk assessment and construction of clear, evidence-based recommendations. Nevertheless, international authorities must provide guidance as a key element of the outbreak response, including advice for international travelers for their own self-protection and in order to limit risks of secondary local transmission on return from travel to areas with the *Aedes* spp. vector.

To develop a broader understanding of the extent of ZIKV among international travelers from Africa, Asia and the Pacific and the role travelers play in identifying areas of endemicity, we analyzed ZIKV infections reported by the GeoSentinel Surveillance Network and juxtaposed these cases with a review of the published literature and web-based reports of ZIKV cases in select countries outside the Americas. We also examined the level and timing of advisories issued by four major international health authorities: WHO, European Center for Disease Control and Prevention (ECDC), US Centers for Disease Control and Prevention (CDC), and Public Health England’s National Travel Health Network and Centre (NaTHNaC).

## Methods

GeoSentinel is a global surveillance network established in 1995 (www.istm.org/geosentinel), currently comprising 64 specialized travel and tropical medicine clinics in 29 countries, the majority being in Europe, America, Australia or New Zealand. To be eligible for inclusion in the GeoSentinel database, patients must have crossed an international border and sought medical advice at a GeoSentinel clinic for a presumed travel-related illness. Data collected include: demographic information, travel data, reason for most recent travel, inpatient or outpatient status, travel duration, and time between the onset of symptoms and presentation at a GeoSentinel clinic. Diagnoses are taken from a list of >500 standardized diagnostic codes. Clinics collect de-identified data on ill travelers for whom they have provided clinical care using a standard form, and report these data to a centralized database [23]. The institutional review board officer at CDC’s National Center for Emerging and Zoonotic Infectious Diseases classifies the data collection protocol as public health surveillance and not human subjects research.

### Data extraction and disease diagnostic criteria

Data on patients diagnosed with ZIKV infection acquired in Africa, Asia or the Pacific (regions defined as per Harvey et al [23]) acquired between 1 January 2012 and 31 December 2016 were extracted from the GeoSentinel database. Sites contributing patient data provided supplemental information including clinical complications and results of diagnostic testing, which was based on local clinician preferences and availability of methods (serology, plaque reduction neutralization test [PRNT], and/or molecular testing including polymerase chain reaction [PCR] and sequencing). Using a modified version of the US Council of State and Territory Epidemiologists (CSTE) case definitions [24] (S1 Table), two clinicians (DHH, MPG) independently classified each patient as confirmed or probable, and discussed discrepancies to reach a final determination. We examined demographic information, itinerary details and reason for travel for each case; country of exposure was based on travel itinerary and relevant incubation periods, with cases excluded if the exposure country could not be ascertained. GeoSentinel cases were considered to be sentinel events (index cases) if no other known human case had been reported in web-based or published literature for at least 2 years prior. Data were managed in Microsoft Excel, with reporting of descriptive statistics.

PubMed, ProMED, International SOS reports, WHO situation reports, ECDC situation reports, the CDC website and online news reports were searched (minimum of two co-authors) using the search criteria “Zika” and “Africa or Asia or Pacific”, or “Zika” and each individual country name in these three regions to identify locally acquired or exported cases. Data on the status and timing of international travel advisories were tabulated following a search of the relevant authorities’ websites, including review of weekly situation reports where relevant [17, 25–27].

## Results

**Fig 1:**
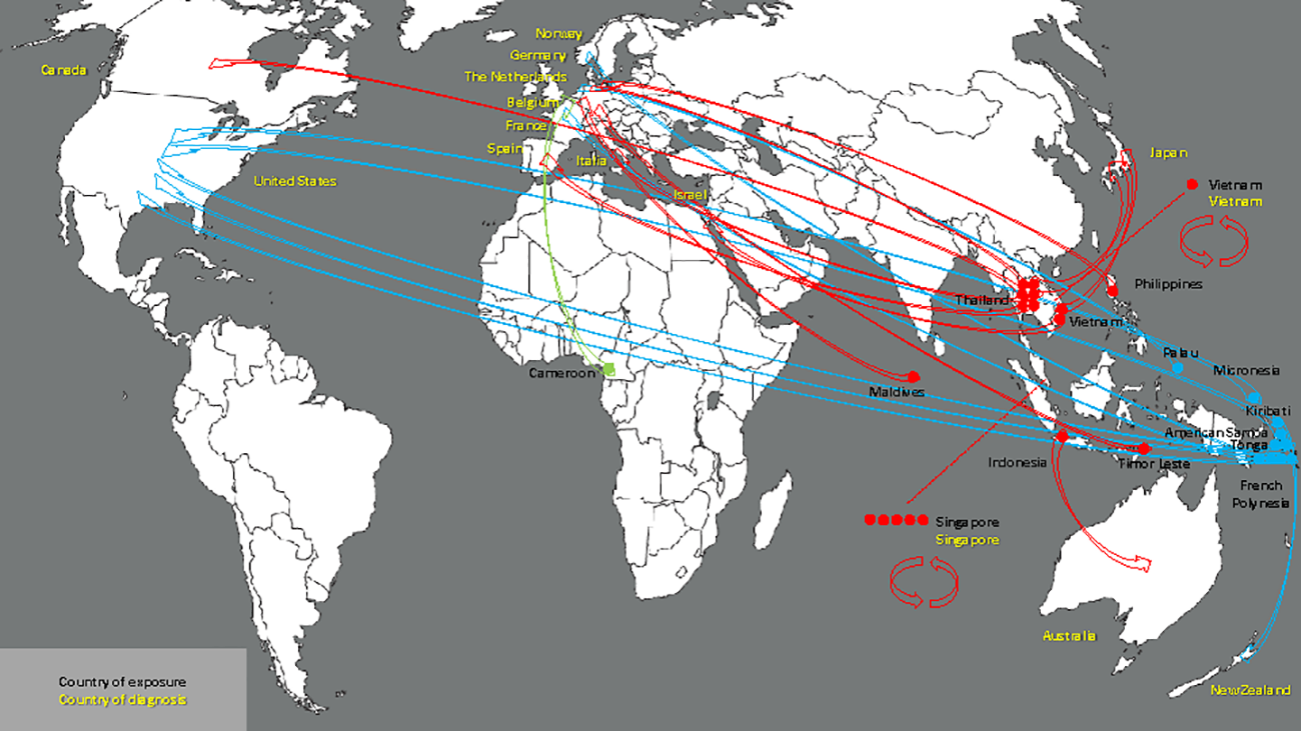
Place of exposure and place of diagnosis (arrows) of GeoSentinel Zika patients from Africa (green dot), Asia (red dots), and the Pacific (blue dots)—Circular arrows indicate cases in international travelers who contracted Zika while abroad and were diagnosed in the country of exposure while traveling. Linear arrows indicate cases in international travelers who contracted Zika while abroad and had their diagnosis confirmed on returning to their home country.—Base map is available free of charge from http://www.histgeo.ac-aix-marseille.fr/ancien_site/carto/.

The first case of ZIKV infection reported to GeoSentinel acquired outside the Americas occurred in a traveler in May 2012 (infection acquired in Indonesia). Since then, two subsequent cases were reported in 2013, five cases in 2014, three in 2015, and 18 cases in 2016 (total of 29 cases, Table 1). Eighteen cases were acquired in Asia, ten in the Pacific, and one in Africa (Fig 1). Twenty-two cases are considered confirmed based on PCR (16 cases), PRNT (3 cases) or serology (5 cases), and seven cases are considered probable. Sixteen cases occurred among males and thirteen among females, one of whom was in the second trimester of pregnancy at the time of exposure and has subsequently given birth to a healthy infant. The median age was 40 years (range: 20–66 years). Fifteen cases occurred among tourists, four among migrant workers, three were travelers visiting friends and relatives, three were missionary workers, two were business travelers, one occurred in a military personnel and one case in an expatriate. Where relevant, the median trip duration to country of exposure was 17 days (range: 6–335 days) and the median time from symptom onset to clinic presentation was 5 days (range: 1–49 days). Twenty-two patients were managed as outpatients. The diagnosis was made at GeoSentinel sites in Asia (8 cases), Western Europe (12 cases), North America (6 cases), Australia/New Zealand (2 cases), and the Middle East (1 case).

**Table 1:** Demographic, itinerary, and laboratory characteristics of GeoSentinel Zika patients from Africa, Asia, and the Pacific.

Country of acquisition	Demographics	Travel Dates	Type of travel and presentation	Diagnosis	Other results	Designation	Comments
American Samoa	26 yo M	Travel: 18 to 25 Jan 2016. Illness onset: 26 Jan 2016	Business travel. Seen after travel as outpatient in US	ZIKV PCR neg but IgM and PRNT pos from specimen taken 29 Feb 2016	Dengue IgM equivocal; dengue PCR and PRNT neg	C	Occurred coincident with earliest cases recognized in American Samoa (1^st^ reports emerged in Jan 2016)
Cameroon	58 yo M	Travel: 23 Feb to 23 Mar 2015. Illness onset: 1 April 2015	Missionary. Seen after travel as inpatient in Belgium	ZIKV IgM pos, ZIKV PRNT >640 from specimen taken on day 7 after illness onset	Dengue serology, NS1 Ag and PCR neg	P	Only reported case of ZIKV in Cameroon since 2010 (also reported in [28]) (incidental retrospective diagnosis while evaluating ZIKV serological diagnostics), but note that probable case only. ***GeoSentinel index case***
French Polynesia (city of Papeete)	32 yo F	Travel: Mid Nov to 5 Dec 2013.Illness onset: 9 Dec 2013	Tourist. Seen after travel as inpatient in Norway	PCR pos from blood specimen taken on day 4 after illness onset (ZIKV IgM and IgG initially neg, but convalescent serum 1 year later was IgM and IgG pos)	Dengue IgM and NS1 Ag neg	C	1^st^ reports of French Polynesian outbreak occurred in Oct/Nov 2013, so not a sentinel event but occurred very early in the outbreak (reported in [29] and [30], ProMED Archive number 20140303.2309965)
French Polynesia	65 yo M	Travel: 27 Dec 2013 to 12 Jan 2014. Illness onset: 7 Jan 2014	Tourist. Seen after travel as outpatient in US	ZIKV IgM (and IgG) and ZIKV PRNT pos from specimen taken on day 7 after illness onset	Dengue IgM also pos but dengue PRNT neg	P	Case coincided with early reports of local outbreak (Nov 2013). Imported into US (likely included in [31]), but note that probable case only
French Polynesia	55 yo M	Travel: 26 Dec 2013 to 7 Jan 2014. Illness onset: 12 Jan 2014	Traveling in military. Seen after travel as outpatient in France	ZIKV IgM and IgG pos from specimen taken on day 16 after illness onset	Dengue serology, NS1 Ag and PCR neg	P	Case coincided with early reports of local outbreak reports (Nov 2013), but note probable case only
French Polynesia	40 yo M	Travel: 16 Oct to 3 Nov 2016. Asymptomatic (screened as wife wanting to conceive). Clinic visit date: 7 Dec 2016	Tourist. Seen after travel as outpatient in France	Zika IgM pos (ELISA) and PRNT pos on blood specimen taken 7 Dec 2016, but PCR from blood and semen neg	Dengue serology not done	P	French Polynesia not considered as having ongoing ZIKV activity in late 2016, but note that probable case only
Indonesia (Jakarta)	52 yo F	14 to 25 May 2012. Illness onset 25 May 2012	Tourist. Seen after travel as outpatient in Australia	PCR pos for flavivirus from blood on specimen taken on day 4 after illness onset sequencing identified ZIKV	Dengue IgM and IgG low pos, NS1 Ag neg, dengue PCR neg	C	Had been reports of positive serosurveys for ZIKV in 1970s and 1980s, but this case (reported in [32]) was the 1^st^ proven case from Indonesia. ***GeoSentinel index case***
Kiribati (South Tarawa)	29 yo F	Travel: Mid -Jan 2015 to 23 April 2015. Illness onset: 25 April 2016	Missionary. Seen after travel as outpatient in New Zealand	PCR pos on blood from specimen taken on day 5 after illness onset	Dengue PCR neg	C	1^st^ reported case of ZIKV ever to be reported from Kiribati. ***GeoSentinel index case***
Maldives	34 yo M	Travel: 3 to 13 June 2016. Illness onset: 16 June 2016	Tourist. Seen after travel as outpatient in Germany	ZIKV PCR pos from blood specimens taken 22 June, 14 July and 26 July and from urine specimen taken 22 July 2016; ZIKV IgM and IgG also pos (IIFAT)	Dengue IgM and PCR neg	C	There are 2 earlier reports of exportation in travelers (June 2015 and Feb 2016) but 1^st^ reports of locally acquired infection occurred in June 2016, so this case imported into Germany coincident with earliest cases recognized in Maldives [30, 33] ProMED Archive Number 20161001.4529740)
Micronesia (Kosrae)	24 yo F	Travel: 1 to 19 Sep 2016. Patient was asymptomatic but presented on return from travel (20 Sep 2016) as was 18 weeks pregnant. Normal obstetric US at 36 weeks (January 2017), declined amniocentesis, gave birth to healthy-appearing baby.	Migrant born in Micronesia, living in the USA, who travelled to Micronesia as a VFR, and was seen after travel as an outpatient in US	ZIKV PCR pos) and IgM pos on blood specimen taken 20 Sep 2016	Dengue IgM and PCR neg	C	Initial reports of outbreak in Kosrae in Jan 2016, with ongoing outbreak activity since (approximately 20 cases per month from Sep to Oct 2016)
Palau	36 yo F	Travel: 26 Sep to 7 Oct 2016. Illness onset: 6 Oct 2016	Tourist. Seen after travel as outpatient in Netherlands	ZIKV IgM and IgG pos on blood specimens taken on day 8 after illness onset, and ZIKV PCR neg on urine	Dengue serology (IgM and IgG) and NS1 Ag neg	P	The first report from Palau of a locally acquired case was on 7 Nov 2016. Symptom onset of this case was one month prior, but diagnosis made coincident with earliest cases recognized in Palau. Note that probable case only
Philippines	20 yo M	Travel: Jan 2014 to 20 Dec 2014. Illness onset: 1 Dec 2014	Missionary. Seen after travel as outpatient in Germany	ZIKV IgM pos 1: 160, ZIKV IgG pos 1: 20480 (IIFT) from blood taken 3 weeks after illness onset	Dengue IgM pos 1: 160, dengue IgG 1:1280. Dengue tests neg when tested in the Philippines during acute illness	C	Positive serosurveys for ZIKV in 1950s in Philippines, and 1^st^ locally acquired case was reported in 2012, but this was 1^st^ case known to have been acquired from Philippines since 2012 (S2 Table). ***GeoSentinel index case***
Singapore	43 yo M	Travel: 9 Aug to >3 Sep 2016. Illness onset: 1 Sep 2016	Tourist from China. Seen during travel while in Singapore as inpatient	ZIKV PCR pos on blood and urine on day 2 after illness onset	Dengue IgM and NS1 Ag neg	C	5 GeoSentinel cases between 28 Aug and 5 Sep 2016 (4 in migrant workers), coinciding with early peak of outbreak
Singapore—locally acquired	25 yo M	Illness onset: 30 Aug 2016	Migrant worker from Bangladesh. Inpatient in Singapore	ZIKV PCR pos on blood and urine on day 3 after illness onset	Dengue IgM and NS1 Ag neg	C	
Singapore—locally acquired	33 yo F	Illness onset: 2 Sep 2016	Migrant worker from Malaysia. Inpatient in Singapore	ZIKV PCR pos (in house assay) on blood and urine on day 3 after illness onset	Dengue IgM and NS1 Ag neg	C	
Presumed acquisition in Singapore	30 yo M	Travel: Visited Malaysia (Ipoh) from 23 to 25 Aug 2016. Illness onset: 27 Aug 2016	Migrant worker from Malaysia. Inpatient in Singapore	ZIKV PCR pos on blood and urine on day 1 after illness onset	Dengue IgM and NS1 Ag neg	C	Presumed to have been acquired in Singapore, although by late Aug/early Sep 2016 cases were being reported from Malaysia, which is notable given travel to Malaysia late Aug 2016 [30] (ProMED Archive number 20160908.4475100)
Singapore—locally acquired	49 yo M	Illness onset: 24 Aug 2016	Migrant worker from Malaysia. Inpatient in Singapore	ZIKV PCR pos on blood and urine on day 5 after illness onset	Dengue IgM and NS1 Ag neg	C	
Thailand	63 yo F	Travel: 20 Jan to 5 Feb 2013. Illness onset: 11 Feb 2013	Tourist. Seen after travel as outpatient in Canada	PCR pos from blood and urine specimen taken on days 1 and 4 after illness onset. ZIKV IgM equivocal, and PRNT pos 15 March (CDC)	Dengue IgM pos but PCR neg	C	Were positive serosurveys from Thailand prior to 2012, but this case (reported in [34]) emerged prior to recent reports of autochthonous transmission (also reported in [30], **ProMED** Archive Number 20130529.1744108). ***GeoSentinel index case***. Subsequent retrospective recognition of sporadic cases (3 locally acquired cases from March 2012, 2 sporadic cases reported in 2013 and 2 cases in 2014 [S2 Table])
Thailand (Koh Samui)	41 yo M	Travel: 25 to 31 July 2014. Illness onset: 2 Aug 2014	Tourist. Seen after travel as outpatient in Japan	ZIKV PCR pos from blood specimen taken on day 2 after illness onset, and from urine taken on day 5; ZIKV IgM also pos (day 5 and day 11 after illness onset)	Dengue IgM pos, NS1 Ag neg	C	2^nd^ GeoSentinel case exported from Thailand (reported in [30, 35], **ProMED** Archive Number 20140823.2716731). Occurred concurrent with early emerging reports of local ZIKV in Thailand
Thailand	25 yo F	Travel: 1 to 31 August 2014. Illness onset: 26 Aug 2014	Tourist. Seen after travel as outpatient in Germany	ZIKV IgM neg but IgG pos > 1:20840 from specimen taken on day 8 after illness onset	Dengue IgM neg, dengue IgG pos 1: 80, and NS1 Ag neg	C	Case not previously reported
Thailand	37 yo M	Travel: 31 July to 18 Aug 2016. Illness onset: unknown. Clinic visit date 23 Aug 2016	Tourist. Seen after travel as outpatient in Italy	ZIKV IgM and IgG neg on 23 Aug and 9 Sep 2016, but PCR pos from urine specimen taken 23 Aug 2016	No dengue serology available	C	Case occurred during known low-level circulation of ZIKV in Thailand. Note that PCR was pos despite neg serology for ZIKV
Thailand	51 yo F	Travel: 5 July to 28 July 2016. Illness onset: 30 Jul. Clinic visit date 9 Aug 2016	Tourist. Seen after travel as outpatient in Spain	ZIKV IgG pos on day 10 after illness onset and on 8 Sep 2016	Dengue IgM neg and PCR neg	C	Case occurred during known low-level circulation of ZIKV in Thailand
Thailand	54 yo F	Travel: 5 July to 28 July 2016. Clinic visit date 9 Aug 2016	Tourist. Seen after travel as outpatient in Spain	Zika IgM and and IgG pos 9 Aug and 8 Sep 2016	Dengue IgM pos and PCR neg	P	Case occurred during known low-level circulation of ZIKV in Thailand. Probable case only
Timor Leste	29 yo M	Travel: 14 to 28 Apr 2016 (brief stop-overs in Bali, Indonesia and in Singapore). Illness onset: 8 May 2016	TouristSeen after travel as outpatient in Germany. (Note: patient presented on 26 June, 2 months after the acute illness when feeling well)	ZIKV IgM pos (IIFT and confirmatory ELISA weakly pos 1.01 [neg <0.8]). ZIKV IgG pos (IIFT pos and confirmatory ELISA pos 1.12 [neg <0.8]) from blood specimen taken 26 June 2016	Dengue IgM and NS1 Ag neg, dengue IgG pos (1:10240)	P	This is the only report of ZIKV from Timor Leste, imported into Germany, but note that probable case only. ***Probable GeoSentinel index case***
Tonga (Nakolo)	66 yo M	Travel: 18 Feb to 3 March 2016. Illness onset: 2 March 2016	VFR. Seen after travel as outpatient in US	ZIKV PCR neg but IgM and confirmatory PRNT pos, titre >20480 on blood specimen taken on day 5 after illness onset	Dengue PCR neg, IgM pos but confirmatory PRNT neg (titre 320)	C	1st cases reported Jan 2016 and 1^st^ exported case in a traveler to Europe reported 10–16 Jan, so these 2 cases imported (husband and wife) occurred relatively early during the local outbreak
Tonga (Nakolo)	60 yo F	Travel: 18 Feb to 3 March 2016. Illness onset: 2 March 2016	VFR. Seen after travel as outpatient in US (wife of patient above)	ZIKV PCR neg but IgM and confirmatory PRNT pos, titre 2460 on blood specimen taken on day 5 after illness onset	Dengue PCR neg, IgM pos but confirmatory PRNT neg (titre <20)	C	As above
Vietnam (Ho Chi Minh City)	61 yo M	Travel: 10 to 23 Dec 2015. Illness onset: 25 Dec 2015	Tourist. Seen after travel as outpatient in Israel	ZIKV IgM and IgG (and ZIKV PCR were pos on blood taken on day 10 after illness onset	Dengue capture IgM, dengue IgG indirect and NS1Ag were neg	C	Case (reported in [30, 36], ProMED Archive number 20160317.4102468) was 1^st^ report of ZIKV from Vietnam since 1954. ***GeoSentinel index case***
Vietnam	49 yo F	Travel: German woman living in Vietnam, traveled to Singapore Aug 16 to 18, and Aug 21 to 22 2016, and then traveled to Japan on 8 Sep and presented on 9 Sep 2016. Illness onset: 6 Sep 2016	Business travel. Seen during travel as an outpatient in Japan	ZIKV PCR pos on urine specimen taken on day 6 after illness onset (neg results from blood on days 3 and 6 after illness onset), , ZIKV IgM pos	Dengue IgM and NS1 Ag neg	C	Case reported in [30] (ProMED Archive number 20160915.4491053)
Vietnam	37 yo F	Illness onset: 19 Dec 2016	Expatriate living in Ho Chi Minh City. Seen as an outpatient in Vietnam	ZIKV PCR (positive on urine on day 1 after illness onset	Dengue IgM and NS1 Ag neg	C	Case occurred during known low-level circulation of ZIKV in Vietnam

Note: no traveller reported any neurological complications. F: female; M: male; yo: year old; ZIKV: Zika virus; pos: positive; neg: negative; PCR: polymerase chain reaction, PRNT: plaque reduction neutralization test; ELISA: enzyme-linked immunosorbent assay; IIFT: indirect immunofluorescence test; C: confirmed, P: probable. Designation of C versus P adapted from [2, 24] (See S1 Table); VFR: visiting friends and relatives

**Fig 2:**
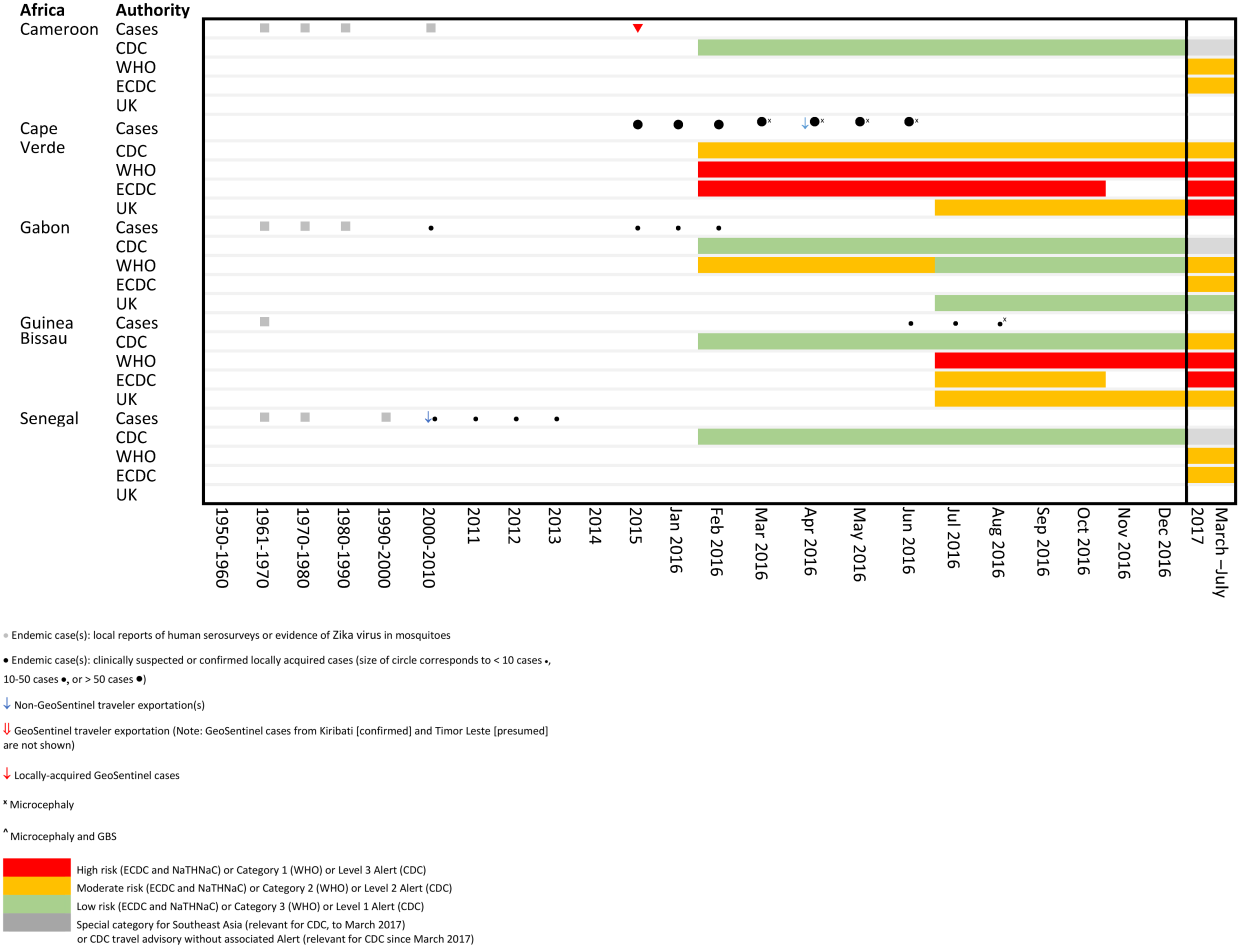
Temporal association of endemic and exported cases with travel notices by international authorities for selected countries in Africa.

**Fig 3:**
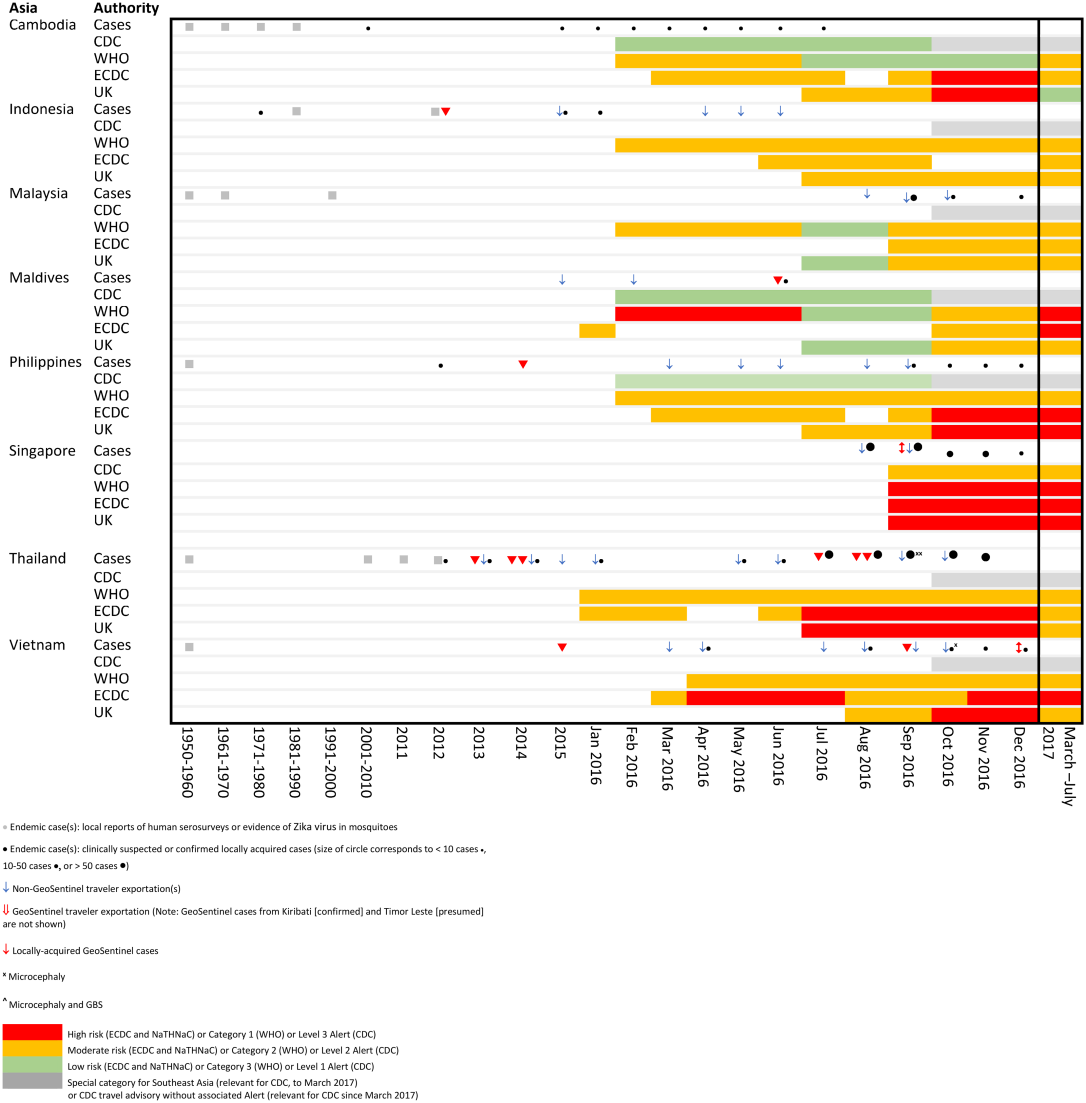
Temporal association of endemic and exported cases with travel notices by international authorities for selected countries in Asia.

**Fig 4:**
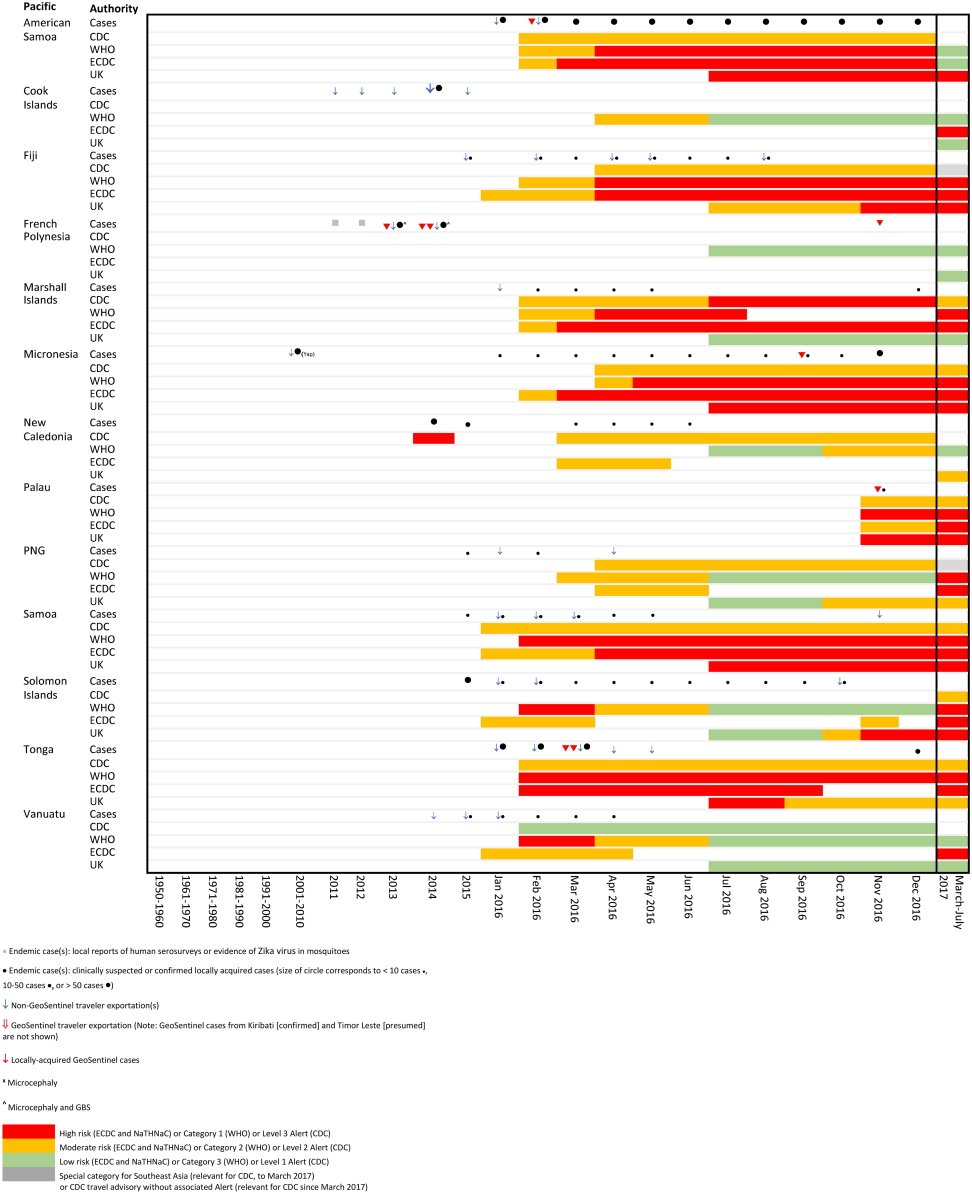
Temporal association of endemic and exported cases with travel notices by international authorities for selected countries in the Pacific.

Comparative temporal reporting of GeoSentinel cases with locally reported cases and exported non- GeoSentinel cases in travelers is shown (Figs 2–4, S2 Table). Eight GeoSentinel cases occurred in travelers who were diagnosed with ZIKV infection following a visit to an area early during a recognized outbreak (within first 4–6 weeks: one case in American Samoa [January 2016], three cases in French Polynesia [December 2013 to January 2014] [29, 31], two cases in Tonga [March 2016], one case in the Maldives [June 2016] [30, 33], and one case in Palau [November 2016]). Five additional cases reported in Singapore similarly presented early after initial outbreak recognition [August to September 2016]. For an additional 7 countries, GeoSentinel cases were the sentinel markers of ZIKV activity, three of which are reported here for the first time (Table 1) [28, 30, 32, 34–36].

- Sentinel case from Indonesia, exported by an Australian traveler in 2012 [32]. Apart from sero-survey data from the 1970s and 1980s (S2 Table), this was the first case from Indonesia (PCR-positive).
- Sentinel case from the Philippines in 2014, exported by a German traveler. This was the first Zika case in the Philippines since 2012 (not reported previously).
- Sentinel, PCR-positive case from Thailand, exported by a Canadian traveler in 2013 [34] (previously serological evidence only from 1950s [S2 Table]).
- Sentinel case from Vietnam, exported by an Israeli traveler in December 2015 [36]. Apart from sero-survey data from the 1950s (S2 Table), this was the first case from Vietnam (PCR-positive).
- Only known case acquired in Cameroon since 2010, exported by a Belgian traveler in 2015 [28].
- First known case reported from Kiribati, exported by a traveler to New Zealand in 2015 (not previously reported).
- Only known case reported from Timor Leste, exported by a German traveler in April 2016 (not previously reported, albeit probable case only).

Comparisons of risk classifications and travel advisories issued by the four major international authorities are shown in Table 2, and are temporally associated with Zika cases as shown in Figs 2–4.

**Table 2:** Risk categorization by major international bodies.

International authority	Risk classifications and travel advisories, 2016	Updated country classifications and advisories since March 2017 (current to 20 May 2017)
**US CDC** [25] (https://wwwnc.cdc.gov/travel/page/zika-information)	1^st^ released interim travel guidelines on 15 Jan 2016, which, “out of an abundance of caution, “advised pregnant women to consider postponing travel to areas with ongoing local transmission, or to take precautions against mosquito bites if they must travel”. **2 categories, Epidemic and Endemic, with 3 levels of travel notices and 2 Alert levels: 1) Epidemic: active transmission**, currently experiencing outbreaks of ZIKV. Travel notices may be posted for these countries, which are considered epidemic because mosquitoes that carry ZIKV are present, ZIKV has not been reported there in the past, and very little, if any, of the population is immune. **Two possible associated travel alert levels associated with epidemic transmission: Level 3 alert**: Avoid non-essential travel; **Level 2 alert**: Practice enhanced precautions. Increased risk in defined settings; certain high-risk populations may wish to delay travel to these destinations. **2) Endemic**: past or sporadic cases (potentially ongoing). Large number of local residents are likely to be immune so the risk to travelers is likely much lower than in epidemic countries, and no travel alert is currently issued.	**Risk levels changed in March 2017, with countries now categorized as** “**Risk of ZIKV”** or “**No known risk**”. Within the two risk areas, the **3 levels of travel notices with 2 categories of Alerts** have been retained: **Level 3 alert**: Avoid non-essential travel; **Level 2 alert**: Practice enhanced precautions (some increased risk so certain high-risk populations may wish to delay travel to these destination); **Level 1**: Watch only, practice usual precautions (limited impact to traveler). Additionally, some countries are listed as having a ZIKV risk but with no alert.
	**Associated travel notice**: Usual or slightly above baseline risk with limited impact to travelers. Watch, practice usual precautions for these destinations. Pregnant women should consult with their health care provider and, if they proceed with travel, strictly follow steps to prevent mosquito bites.	All countries designated as having a ZIKV risk have a **travel recommendation** such that pregnant women should avoid travel to these areas. CDC classifies areas with a risk of Zika virus according to WHO Categories 1 and 2, plus a subgroup of Category 4 countries (those that share a border and ecologic zone with another country which has endemic transmission [Category 4a countries as defined by ECDC]). Countries classified as no risk correspond to WHO Categories 3, 4-non-subgroup (Category 4b), and countries without a competent vector.
	**Exception: “Special considerations**” notice issued for 11 Southeast Asian countries on September 30^th^ 2016 [38]: Countries in Southeast Asia with previously reported ZIKV or case exportations, but with limited data available to fully evaluate risks, were considered to have endemic disease with large numbers of immune local residents. Risks to non-immune travelers were thought to be lower than in areas where ZIKV is newly introduced and spreading widely. Travel notices were **not** issued for these destinations but pregnant women were advised to consult with health care providers and consider postponing non-essential travel	The Special Considerations notice for Southeast Asia was removed in March 2017, and most countries previously listed in this group were incorporated into those with ZIKV risk but with no associated Alert.
**WHO** [17, 39] (see http://www.who.int/csr/disease/zika/information-for-travelers/en/ and http://www.who.int/csr/resources/publications/zika/classification/en/)	Numerous changes in WHO categories between 1^st^ warnings on 5 Feb 2016 and 14 April 2016, but generally captured 3 categories: i) Reported autochthonous transmission; ii) Indication of viral circulation; iii) Countries previously affected by ZIKV transmission/outbreaks terminated. **Categories changed on 14 April 2016 to 7 July 2016: Category 1**. Countries experiencing a first outbreak of ZIKV since 2015, with no previous evidence of circulation, and with ongoing transmission by mosquitos. **Category 2**. Countries with evidence of ZIKV transmission prior to 2015, with or without ongoing transmission; or countries where outbreak is over. From 7^th^ July 2016 to March 2017, **3 risk category levels were in place, with travel advisory issued for Categories 1 and 2: Category 1: Countries with reported outbreak from 2015 onwards**. Laboratory confirmed, autochthonous, mosquito-borne case of ZIKV infection in an area with no evidence of circulation of the virus in the past (prior 2015), **OR** in an area where transmission has been previously interrupted (assumes that there is sufficient population susceptibility to allow transmission);**OR** Increase in incidence of laboratory confirmed, autochthonous, mosquito-borne ZIKV infection in areas where there is on-going transmission (> two standard deviations of baseline rate, or doubling of case numbers over a 4-week period). **Category 2: Countries with possible endemic transmission or evidence of local mosquito-borne Zika infections in 2016**. Countries reporting an outbreak with consistent presence of confirmed, autochthonous, mosquito-borne cases of ZIKV infection 12 months after the outbreak **OR** Countries where ZIKV has been circulating for several years with consistent presence of confirmed, autochthonous, mosquito-borne cases of ZIKV infection or evidence of local mosquito-borne ZIKV in 2016. **Category 3: Countries with evidence of local mosquito-borne Zika infections in or before 2015, but without documentation of cases in 2016, or outbreak terminated**. Absence of confirmed cases over a 3-month period in areas with climatic conditions suitable for year-round arbovirus transmission, or over a 12-month period in areas with seasonal vector activity	**In March 2017, WHO reclassified countries into 4 epidemiologic Categories, with travel advisory issued for Categories 1 and 2: Category 1: Area with new introduction or re-introduction with ongoing transmission**. Laboratory confirmed locally acquired case in either local population or in a traveller returning to a second country. **Category 2: Area either with evidence of virus circulation before 2015 or area with ongoing transmission that is no longer in the new or re-introduction phase, but where there is no evidence of interruption**. Includes countries with known historical laboratory evidence of ZIKV circulation prior to 2015, based on the literature or on surveillance data (whether detected and reported by the country where infection occurred or by another country reporting a confirmed case in a returning traveler). Countries in this category may have seasonal variations in transmission. These countries may also experience outbreaks of ZIKV disease. **Category 3: Area with interrupted transmission and with potential for future transmission**. The minimum timeline for determining transition to an interrupted state is 12 months after the last confirmed case, and no cases identified in travelers. For countries with a high capacity for diagnostic testing, consistent timely reporting of diagnostic results, a comprehensive arboviral surveillance system and/or a temperate climate or island setting, the interruption of vector-borne transmission is defined as the *absence of ZIKV infection 3 months after the last confirmed case*. **Category 4: Area with established competent vector but no known documented past transmission or current transmission**. All areas where the main competent vector (A. aegypti) is established, but which have not had a documented, autochthonous, vector-borne case of ZIKV infection. This category also includes areas where ZIKV transmission may occur because of a shared border with a neighbouring Category 2 country, by belonging to the same ecological zone and having evidence of dengue virus transmission. In this subgroup, a first laboratory-confirmed, autochthonous vector-borne case of ZIKV infection may not necessarily indicate new introduction (Category 1), but rather previously unknown and undetected transmission (Category 2).
	**Associated travel advisory for categories 1 and 2**: WHO advises pregnant women not to travel to areas with ongoing ZIKV outbreaks because of increased risk of microcephaly and other congenital malformations. Healthcare practitioners advising travelers should: i) Provide travelers to areas with ongoing ZIKV outbreaks with up-to-date advice on reducing infection risks (preventing mosquito bites and practicing safer sex); ii) Advise travelers to practice safer sex or consider abstinence for at least 6 months and not to donate blood for at least 1 month after return; iii) Advise pregnant women not to travel to areas with ongoing ZIKV outbreaks; iv) Advise pregnant women whose sexual partners live in or travel to areas with ongoing ZIKV outbreaks to ensure safer sexual practices or abstain from sex for the duration of their pregnancy	**Categories 1 and 2 are associated with travel advisory**: Pregnant women should not to travel to these countries
**European CDC** [26, 40] (see http://ecdc.europa.eu/en/healthtopics/zika_virus_infection/zika-outbreak/Pages/Zika-countries-with-transmission.aspx and http://ecdc.europa.eu/en/publications/Publications/21-03-2017-RRA%20UPDATE%209-Zika%20virus-Americas,%20Caribbean,%20Oceania,%20Asia.pdf)	1^st^ issued notices on 21 Jan 2016. Initially determined if country was “affected”, then changed to listing according to “autochthonous in prior 9 months” or “prior 2 months”, then changed to **two risk categories with 2 corresponding levels of travel advisories. Widespread transmission**: > 10 locally transmitted cases of ZIKV in one area, OR local transmission of ZIKV in two or more areas, OR ZIKV transmission ongoing for > 3 months; **Sporadic transmission**: no more than 10 locally transmitted cases reported in a single area in past 3 months	**Risk categories changed in April 2017 to 4 categories of risk (corresponding to the WHO categories listed above) plus two additional subcategories: 1)** Areas with virus transmission following virus new/re introduction (corresponds to WHO Cat. 1); **2a)** WHO category 2 areas with virus transmission following previous virus circulation; **2b)** WHO category 2 subgroup: areas with new documented intense transmission (10 or more confirmed/probable/suspected cases documented in last three months, or two or more confirmed/probable/suspected cases documented in the last three months in at least two locations); **3)** Areas with interrupted transmission (corresponds to WHO Cat.3); **4a**: Areas bordering a WHO category 2 area, indicating a higher risk of transmission because of the proximity with a category 2 area, sharing the same ecological characteristics or experiencing virus transmission following past virus circulation (sub-category of WHO Cat. 4). Other countries and areas have been listed as category 4b; **4b**: Areas with potential for transmission
	**Travel notices: For widespread transmission**, all travelers to affected areas are at risk of ZIKV infection unless they have immunity due to a previous infection. Pregnant women and travelers with immune disorders or severe chronic illnesses should seek pre-travel advice and postpone non-essential travel. Women of childbearing age who travel to affected areas should take measures to prevent mosquito bites and follow recommendations for prevention of sexual transmission while in affected areas. **For sporadic transmission**, all travelers to affected areas are at risk of ZIKV infection unless they have immunity due to previous infection. Pregnant women should seek pre-travel advice and consider postponing non-essential travel. Travelers should take measures to prevent mosquito-borne and sexual transmission of ZIKV.	**Category 1** and **Category 2 intense transmission subgroup** are considered high risk; **Category 2** is considered moderate risk, **Category 4a** is considered low risk. **Categories 3 and 4b** are considered very low risk and have no associated travel advisory.
**Public Health England/NaTHNaC** [27] (https://www.gov.uk/guidance/zika-virus-country-specific-risk)	1^st^ published warnings 25 July 2016. **Four risk categories for ZIKV transmission: High, moderate, low and very low risk, with 4 corresponding travel advisories. High risk (active transmission in the last 3 months)**: Subcategories: High risk (a): All countries that have reported active and increasing or widespread ZIKV transmission in past 3 months or High risk (b): Countries within main outbreak regions that have reported active but sporadic ZIKV transmission in past 3 months. **Moderate (sporadic transmission in the last 6 months)**: Subcategories: Moderate risk (a): Countries outside main outbreak regions reporting active but sporadic ZIKV transmission in past 6 months or Moderate risk (b): Countries within main outbreak regions but no recorded cases in past 6 months. **Low (no cases in the last 3 months)**: Countries with evidence of recent ZIKV transmission since 2007 but no recorded cases in past 6 months. **Very Low (Zika prior to 2007 only)**: Countries with historical evidence of ZIKV transmission (pre-2007)	**No change between Dec 2016 and Aug 2017. (Note: risk categories changed in Aug 2017 to better correlate with WHO and ECDC**. There are still are 4 risk categories: **high, moderate, low or very low risk.)**
	**Travel advisories: High risk**: Pregnant women should postpone non-essential travel; **Moderate risk**: Pregnant women should consider postponing non-essential travel; **High and moderate risk**: All travelers to high and moderate risk countries should be given advice on ZIKV prevention, including avoidance of mosquito bites and sexual transmission; **Low risk**: Individual risk assessment advised pre-travel to discuss low risk; **Very low risk**: No advisory	

CDC: US Centers for Disease Control and Prevention (CDC); ECDC: European Center for Disease Control and Prevention; WHO: World Health Organisation; NaTHNaC: National Travel Health Network and Centre (NaTHNaC), Public. Note: As of April 2017, countries with a Level 2 alert per CDC largely correspond to the Category 1 list (new introduction or re-introduction and ongoing transmission). Other countries listed as having ZIKV risk per CDC (with corresponding advice for pregnant women to avoid travel to these areas) but with no Level 2 alert correspond to Category 2 (past or ongoing transmission) or Category 4 subgroup (competent vector exists, no past or current transmission, but bordering a WHO category 2 area). By contrast, WHO has no travel advisory for Category 4 countries. Correlation with NaTHNaC categories, which additionally, provides a level of risk intensity, has been somewhat variable, although in Aug 2017 NaTHNaC updated the categories to be more in line with WHO and ECDC

## Discussion

Travelers are key to the global spread of ZIKV [37]. This report emphasizes the vital role travelers play as sentinels, the complementary information systematic traveler surveillance provides to local surveillance data, and the role travelers play in understanding the geographic extent of virus circulation in areas that may otherwise be undetected because of often-asymptomatic infections, limited surveillance, sub-optimal diagnostics, and co-circulation of other arboviruses [2, 7, 36, 41]. When returning home to countries where competent vectors are present, travelers can introduce ZIKV into new areas, thereby also providing a source for propagation and circulation of infection among the local population [18, 42].

The GeoSentinel ZIKV case acquired in Indonesia in May 2012 was among the first sporadic cases reported in a traveler [32, 36]. In countries with lower access to reliable diagnostic testing, local surveillance systems may have limited capacity to detect endemic ZIKV activity, and patient assessment in well-resourced sites facilitates high-level diagnostic support from reference laboratories. Limited awareness of ZIKV may have resulted in earlier missed cases, but since then travelers have been involved in many additional importations globally, with exportations from Asia, the Pacific and Africa highlighted here since GeoSentinel cases from the Americas were recently reported [2].

The role travel-associated cases, including those reported by GeoSentinel, have played as indicator of local ZIKV infections has varied in different regions. ZIKV in South America, the Caribbean and the Pacific led to clinically apparent local outbreaks, with exported cases in travelers also reported [2]. GeoSentinel data cannot be used to infer risk of infection since there is non-uniformity in the likelihood of presenting to a specialized GeoSentinel clinic according to geographical and other factors, but as of the end of 2016, over 400 cases of ZIKV had been reported to GeoSentinel following travel to the Americas, compared to the 29 cases reported here from other regions. Comprehensive seroprevalence and strain cross-reactivity studies are lacking, but there appears to be lower epidemic potential for ZIKV in most African and Asian countries, with fewer recognized local cases and exported cases not infrequently triggering recognition of ZIKV circulation [16]. For Africa, exported cases in travelers have been reported from Senegal [43] and Cape Verde [33], in addition to the GeoSentinel case from Cameroon [28]. For Asia, we are aware of only a single country (Malaysia) that has reported exported cases but for which there is not a confirmed GeoSentinel case. Moreover, GeoSentinel cases served as the first identification of recent ZIKV activity in all Asian countries with known circulation except for the Maldives and Singapore, highlighting the importance of travelers in understanding ZIKV epidemiology. The lower epidemic potential particularly in Asia is presumed to be because of long-standing lower-level endemic ZIKV circulation, prior exposure to a broad range of flaviviruses and prior immunization with Japanese encephalitis vaccine which potentially provides cross-protection to ZIKV [44]. These factors which may have resulted in higher background ZIKV seroprevalence, greater levels of population-wide immunity, fewer clinical manifestations, and greater difficulties with serological confirmation [7].

During 2016, there were significant differences in risk categorizations and in interpretation of data by the four major international authorities, possibly underpinned by political factors, which created marked variability in country stratifications and non-uniformity in both timing and presence of travel advisories (Figs 2–4, S2 Table). While travel advisories generally presume that the presence of endemic disease and a partially immune population pose less risk to travelers than travel during a recognized outbreak, the impact on transmission risks to non-immune visiting travelers from varying levels of herd immunity in local communities remains uncertain. In March 2017, country classifications for ZIKV epidemiology were modified by a working group formed by the WHO, CDC and ECDC in order to achieve greater harmonization, with resulting revisions leading to four main categories (Category 1: new or re-introduction and ongoing transmission; Category 2: past or ongoing transmission but not in the new or re-introduction phase; Category 3: interrupted transmission with potential for future transmission; Category 4: competent vector exists but no known past or current transmission, Table 2 [39]). However, each organization handles risk mitigation differently: CDC refers to the four country classification designations by WHO [45], but lists only two broad transmission risk levels: “risk present” and “risk absent” [25]. ECDC has the same four Zika virus epidemiology classifications as WHO, but differentiates the WHO Category 2 countries with current outbreaks, and also shows Category 4 subgroups [40]. NaTHNaC has four risk categories, with risk levels ranked into high, medium, low, or very low risk [27] (Table 2). Furthermore, there is non-uniformity in correlation of country classifications with travel advisories. CDC, ECDC and WHO all make recommendations for pregnant women to avoid travel to countries with documented ZIKV transmission in Categories 1 and 2. CDC further recommends avoiding travel to Category 4a subgroup countries. ECDC differentially grades the risk both for Category 2 countries (endemic countries versus endemic countries with documented “intense transmission”), and for the two Category 4 countries (Category 4a subgroup countries versus 4b). WHO provides no ZIKV warning for those travelling to countries in the Category 4 sub-group. Correlation with NaTHNac categories is somewhat variable. The result is a complex, inconsistent set of recommendations where assessment of the travel risks to pregnant travelers for some specific destinations may be listed anywhere from no risk, caution only or high risk for which travel should be avoided depending on the source of information sought. For example, for Kenya and Tanzania, CDC recommends avoidance of travel, whereas other agencies have no risk warning (as of September 2017).

In addition to uncertainties on ZIKV epidemiology, case definitions vary. The current CSTE definitions [24] are more detailed than WHO definitions [46], but two aspects of these definitions that merit attention. First, neither addresses anti-Zika IgG (likely because of assay unavailability in the United States), so in our modified case definitions we have added mention of IgG to both probable and confirmed categories (S1 Table). The more challenging limitation relates to interpretation of cases, such as the importation from Cameroon, for which dengue serology was negative (as were NS1 Ag and PCR), yet Zika IgM and confirmatory PRNT were positive. When routine tests for dengue serology are negative and Zika serology is positive, many centers would not perform PRNT. Furthermore, cases with positive PCR and negative serology are now recognized (such as the GeoSentinel case exported from Thailand to Italy, Table 1), leading to additional complexities with defining optimal testing algorithms. Updated guidelines for global definitions which take into account varying scenarios, optimal testing according to timing of presentation relative to exposure, and assay availability in different countries are needed.

Characteristics of ZIKV transmission can often be best determined in travelers returning from endemic areas, for which the period of exposure may be more easily determined than among local populations. Sexual transmission is best assessed in non-endemic settings where there are no competent mosquito vectors; notably the first detection of sexual transmission occurred in a traveler from Africa [43]. Additionally, investigation of returned travelers has ascertained the maximum reported period between symptom onset and sexual transmission (34–41 days) [47], the longest duration of shedding of infectious virus particles (69 days) [48], and the maximum duration of detection of Zika genomic sequences in semen (188 days) [49]. These data underpin current international guidelines recommendations for avoidance of pregnancy for at least 6 months after potential exposure for males and at least 8 weeks for females.

A limitation of this descriptive report is that surveillance data captured by GeoSentinel include only cases seen at specialized clinics, mostly in developed countries; they do not capture all imported cases and may not be representative of all global travelers. Moreover, GeoSentinel is unable to provide denominator data or absolute risk information. However, GeoSentinel’s deliberate focus to collate data from highly specialized clinics underscores the utility of assessing disease in travelers as a mechanism for identifying geographic areas where emerging diseases may be occurring. Another limitation is that we cannot entirely exclude diagnosis or (unpublished) recognition of additional earlier ZIKV cases, thereby potentially overstating the value of the GeoSentinel reports as index cases. However, given our extensive review of published data and media sources, we nevertheless assert that GeoSentinel reports provide important additive case information.

In summary, many uncertainties in ZIKV epidemiology, transmission and risk of adverse outcomes remain, and consensus on diagnostic and definition issues is needed. We have described ZIKV cases among travelers visiting Africa, Asia and the Pacific, highlighted the integral role of travelers as sentinels of ZIKV infection, shown that GeoSentinel assists in the identification of these travelers, and have contextualized GeoSentinel cases with other reported (locally acquired or exported) cases. We have also demonstrated the differential impact of travelers as sentinels in Asia, which likely relates to the lower epidemic potential due to prior flavivirus circulation among the population of this region. Our findings also detail the basis for non-uniformity in travel advisories issued by the four major international authorities. The presence of variable travel advisories may create confusion for health practitioners and individual travelers when determining risks, and the small but non-zero potential for causing devastating fetal outcomes must be discussed. Traveler consultations need to account for overall ZIKV risk assessment, residual risk uncertainties and individual risk tolerance.

## Supporting information

S1 Table Definition of clinically suspected and confirmed cases used in the current series (adapted from US Council of State and Territory Epidemiologists Interim Zika virus Disease case definition [CSTE][2, 24]). (DOCX)

S2 Table Reports of endemic Zika virus (ZIKV) cases, exported cases, and major travel authority notices during the height of the Zika outbreak (to Dec 2016). (DOCX)
