# Supplementary material for: Zika beyond the Americas: Travelers as sentinels of Zika virus transmission. A GeoSentinel analysis, 2012 to 2016
Source: PLoS One. 2017 Oct 3;12(10):e0185689. doi: 10.1371/journal.pone.0185689 (PMC5626466; doi:10.1371/journal.pone.0185689)
Supplement: S1 Table — (DOCX) [file pone.0185689.s001.docx]

**S1 Table: Definition of clinically suspected and confirmed cases used in the current series (adapted from US Council of State and Territory Epidemiologists Interim Zika Virus Disease Case Definition [CSTE]^1,2^)**

| **Clinical Criteria**  A person with one or more of the following:   - - acute onset of fever (measured or reported)   - maculopapular rash   - arthralgia   - conjunctivitis   - complication of pregnancy     - fetal loss in a mother with compatible illness and/or epidemiologic risk factors; or     - *in utero* findings of microcephaly and/or intracranial calcifications with maternal risk factors   - Guillain-Barré syndrome not known to be associated with another diagnosed etiology |
| --- |
| **Probable case**  Meets clinical criteria AND   - - resides in or has recently traveled to an area with ongoing Zika virus transmission, OR   - has direct epidemiologic linkage to a person with laboratory evidence of recent Zika virus infection (e.g. sexual contact, in utero or perinatal transmission, blood transfusion, organ transplantation), OR   - association in time and place with a confirmed or probable case   AND meets the following laboratory criteria:   - - positive Zika virus-specific immunoglobulin M antibodies in serum or cerebrospinal fluid (CSF); or positive Zika virus-specific IgG antibodies in serum and   - negative dengue virus-specific immunoglobulin M or IgG antibodies; AND     - no neutralizing antibody testing performed; or     - less than four-fold difference in neutralizing antibody titers between Zika virus and dengue or other flaviviruses endemic to the region where exposure occurred |
| **Confirmed case**  Meets clinical criteria AND  Has laboratory evidence of recent Zika virus infection by:   - - detection of Zika virus by culture, viral antigen or viral ribonucleic acid in serum, CSF, tissue, or other specimen (e.g. amniotic fluid, urine, semen, saliva); OR   - Zika virus IgM antibodies in serum or CSF **with** Zika virus neutralizing antibody titers 4-fold or greater than neutralizing antibody titers against dengue or other flaviviruses endemic to the region where exposure occurred   - Positive Zika virus-specific IgG antibodies in serum **with** Zika virus neutralizing antibody titers 4-fold or greater than neutralizing antibody titers against dengue or other flaviviruses endemic to the region where exposure occurred |
