# Supplementary material for: Zika beyond the Americas: Travelers as sentinels of Zika virus transmission. A GeoSentinel analysis, 2012 to 2016
Source: PLoS One. 2017 Oct 3;12(10):e0185689. doi: 10.1371/journal.pone.0185689 (PMC5626466; doi:10.1371/journal.pone.0185689)
Supplement: S2 Table — (DOCX) [file pone.0185689.s002.docx]

**S2 Table: Reports of endemic Zika virus (ZIKV) cases, exported cases, and major travel authority notices during the height of the Zika outbreak (to Dec 2016)**

|  | **Year of report (Prior to 2016)** | **2016 endemic reports** | **Exported cases** | **Travel notices**  **CDC^3^** | **Travel notices**  **WHO^4^** | **Travel notices ECDC^5^** | **Travel notices**  **NathNac**^6^ |
| --- | --- | --- | --- | --- | --- | --- | --- |
| Africa | | | | | | | |
| Burkina Faso | **1963-1983**: serological surveys and evidence of ZIKV presence in mosquitoes^7-9^ |  |  | Endemic, risk to travelers is low | No risk (not listed) | No risk (not listed) | No risk (not listed) |
| Cameroon | **1960-1983**:serological surveys suggest possible evidence of ZIKV presence^7,8,10,11^  **1964:** ZIKV isolated from mosquitoes^12^  **2010**: Suggestive ZIKV antibodies in human blood^13^ |  | **23 March 2015:** Retrospective diagnosis of a case in a man who returned to Belgium, blood taken 8 April 2015 (reported in^14^, ***GeoSentinel case and sentinel marker of disease activity***)* | Endemic, risk to travelers is low | No risk (not listed) | Past transmission | No risk (not listed) |
| Cape Verde | **Sep 2015:** Reports of cases of ZIKV-like illness. By **14 Oct**, 165 suspected cases are reported  **21 Oct 2015:** PCR confirmation of Cape Verde’s first outbreak of ZIKV infection (subsequently confirmed to be American variant of ZIKV imported from Brazil, so ***example of travelers as “vectors” of disease***), 165 suspected cases reported^15#^  **6 December 2015:** 4744 suspected cases, 17 confirmed^16^ (Archive number 20151223.3886435) | **1^st^ ECDC report Jan 29 2016:** Between end of Sep and 6 Dec 2015, Cape Verde reported 4744 suspected cases of ZIKV infection, peaking end Nov then declining^17^  **1 Feb 2016:** 7081 suspected cases^18^  **14 March 2016:** microcephalic baby suspected linked to ZIKV infection^18^  **11 April 2016**: testing of > 800 people for ZIKV showed IgM in 200, with > 100 pregnant women testing positive^18^  **20 May 2016:** In total, >7550 cases reported since Sep 2015. Brazilian strain of ZIKV confirmed to have caused the outbreak. Notable as 1^st^ identification of this strain in Africa^18^  **10 June 2016**: 6 cases of microcephaly and other neurological abnormalities in cases with previous ZIKV infection^18^  **28 Nov 2016**: total of 9 cases of microcephaly | 2 cases imported into Europe, most recent **17-23 April 2016**^19^ | **22 Jan 2016 to Dec 2016:** Epidemic, Level 2 travel warning | **5 Feb 2016**: autochthonous transmission  **14 April 2016:** outbreak  **7 July:** Category 1 | **Affected in 2015^17^**  **Jan 19 2016**: reported confirmed autochthonous cases in past 2 months  **29 Jan to 14 Oct 2016**: Increasing or widespread  **21 Oct to Dec 2016:** not listed | **22 Jan 2016**: Epidemic, Level 2 travel warning |
| Central African Republic | **1961**: serologic evidence of human ZIKV infection^20^  **1961-2**: evidence of ZIKV infection in mammals^20^  **1979**: serosurvey showed high levels of seropositivity in the human population^21^. Also isolated from mosquitoes^9^  **1976-1980**: isolation of ZIKV from mosquitoes^22^ |  |  | Endemic, risk to travelers is low | No risk (not listed) | No risk (not listed) | No risk (not listed) |
| Cote d’Ivoire | **1963-65**: 1961: serologic evidence of human ZIKV infection^7,23^  **1999**: evidence of ZIKV in mosquitoes in Cote d’Ivoire^24^ |  |  | Endemic, risk to travelers is low | No risk (not listed) | No risk (not listed) | No risk (not listed) |
| Egypt | **1953**: report of serologic surveys suggesting evidence of human ZIKV infection^25^ |  |  | Endemic, risk to travelers is low | No risk (not listed) |  | No risk (not listed) |
| Gabon | **1967**: serologic evidence of human ZIKV infection^7^  **1975**: serologic evidence of human ZIKV infection^26^  **1982**: serologic evidence of simian ZIKV infection reported^27^  **2007-2010**: first direct evidence of human ZIKV infection circulating in Gabon, and its first occurrence in *Aedes albopictu*s^9^  **2015**: sporadic local cases | **Early 2016:** ongoing low-level transmission suspected |  | Endemic, risk to travelers is low | **5 Feb 2016**: viral circulation 14 April 2016: past transmission, with or without ongoing transmission **7 July to Dec 2016**: Category 3 | Past transmission | Low risk |
| Guinea-Bissau | **1964-65**: serologic evidence of human ZIKV infection^7,28^ | **29 June 2016**: first cases ZIKV reported (3 cases confirmed on Bijagós archipelago)^18^  **August 2016:** 5 suspected cases of microcephaly reported (preliminarily confirmed that the cases are of the African lineage) |  | Endemic, risk to travelers is low | **7 July 2016 to Dec 2016:** Category 1 | **From 8 July until 14 Oct 2016:** Listed as sporadic transmission | Moderate risk |
| Nigeria | **1951**: ZIKV identified in serologic study in children^29^  **1953**: 3 clinical cases of ZIKV, including the first isolation of ZIKV from a young girl with fever and headache^30^  **1964 to 1970**: 3 clinical cases of human ZIKV infection^7,31^  **1971-1975**: two ZIKV isolations from human cases of mild febrile illness, and 40% seropositivity among Nigerians^32,33^  **1980**: serosurveys show evidence of human ZIKV infection^34^ |  |  | Endemic, risk to travelers is low | No risk (not listed) | No risk (not listed) | No risk (not listed) |
| Senegal | **1962**: serologic survey suggested presence of human ZIKV infection^7^  **1967-8**: ZIKV detected in wild mammals^7^  **1972**: ZIKV antibodies found in human samples^35^  **1972-75**: serologic evidence of human ZIKV infection^35^. Also isolated from mosquitoes^9^  **1990:** human strain of ZIKV isolated^36^  **2009-2013**: 9 locally-acquired ZIKV cases detected^37^  **2011**: ZIKV discovered in mosquitoes^38^ |  | **2008**: Exported to USA by 2 scientists doing field work in Senegal. Notable as one case was the 1^st^ report of presumed sexual transmission of ZIKV (one case infected his wife)^39^ | Endemic, risk to travelers is low | No risk (not listed) | Past transmission | No risk (not listed) |
| Sierra Leone | **1972**: serologic survey suggested presence of human ZIKV infection^40^ |  |  | Endemic, risk to travelers is low | No risk (not listed) | No risk (not listed) | No risk (not listed) |
| Tanzania | **1952:** 1^st^ human cases detected in serological study^41^  **1980’s**: further detection of cases in humans |  |  | Endemic, risk to travelers is low | No risk (not listed) | No risk (not listed) | No risk (not listed) |
| Uganda | **1947:** Scientists identify new virus in rhesus monkey in Zika forest of Uganda, named as the **Zika** virus^42^  **1948**: ZIKV isolated from Aedes africanus mosquitoes trapped in Zika forest^41,42^  **1952:** 1^st^ human cases detected in serological studies with seroprevalence of 6.1%^41,43^  **1958:** ZIKV strains isolated from Aedes africanus mosquitos from Zika forest area^44^  **1961-3**: isolation of ZIKV from mosquitoes^45^  **1962**: A researcher in Uganda is infected with ZIKV while working on the virus, developed a mild illness, and confirmed ZIKV causes human disease^46^  **1966-7**: serologic evidence of human ZIKV infection^47^  **1969-70**: epizootics in monkeys^48^  **1984**: ZIKV antibodies found in human blood sample^49^ |  |  | Endemic, risk to travelers is low | No risk (not listed) | No risk (not listed) | No risk (not listed) |
| Other | **1950’s-1970s**: positive antibody results in serological studies: Angola^50,51^, Benin^7^, Ethiopia^52^, Kenya^47,53^, Liberia^7^, Mali^7^, Morocco^7^, Mozambique^54,55^, Niger^7^, Somalia^47^, Togo^7^  **2014**: Zambian serosurvey suggested exposure to ZIKV in 6.1% of the population^56^ |  | **Dec 15^th^ 2016**: traveler from Angola to France presented with illness compatible with ZIKV infection. Previous yellow fever vaccination and sero-positivity for other flaviviruses precluded conclusive diagnosis of ZIKV^4^ | CDC lists the following countries as endemic, low risk to travelers: Angola, Benin, Ethiopia, Kenya, Liberia, Mali, Mozambique, Niger, Samolia, Togo, Zambia  (Morocco – not listed) |  |  |  |
| Asia | | | | | | | |
| Bangladesh | **2014:** Retrospective study identified a case in a 67 year old man from blood taken in 2014 (reported March 2016)^16^ (Archive Number 20160323.4112454 and 20160325.4118019) |  |  | Endemic, risk to travelers is low | **17 March 2016:**  autochthonous transmission 14 April 2016: past transmission, with or without ongoing transmission **7 July to Dec 2016**: Category 3 |  | Low risk |
| Cambodia | **1951 through 1982:** mentioned in WHO timeline^8^  **2007-2010:** cases of locally acquired ZIKV infection^57^  **2007-2015**: Retrospective study found cases by PCR in 2007, 2008, 2009 and 2015^58^ | **11 Nov 2016**: 1^st^ case of ZIKV since 2010 in a 44-year-old man (8th case since 2007 in Cambodia), but subsequently corrected by [Cambodian Ministry of Health](http://www.cdcmoh.gov.kh/) as testing was negative)^18^  **Dec 2016:** Retrospective study from patients and mosquitoes (samples taken 2007-2016) suggesting low prevalence ZIKV^~~58~~^ |  | Endemic, risk to travelers is low  **30 Sep 2016:** Included in CDC’s Southeast Asia Regional special consideration notice | **5 Feb 2016**: viral circulation 14 April 2016: past transmission, with or without ongoing transmission **7 July to Dec 2016:** Category 3 | Past transmission | Low risk |
| India | **1952:** serologic evidence of human ZIKV infection reported (seroprevalence of ZIKV antibodies 16.8%)^59^ |  |  | Endemic, risk to travelers is low | No risk (not listed) |  | No risk (not listed) |
| Indonesia | **1977-78**: ZIKV identified as cause of fever in 7 hospitalized cases in central Java, Indonesia^60^, representing 1^st^ confirmed human cases outside Africa  **1983:** serological study reported suggesting evidence of human ZIKV infection^61^  **2012:** positive serological test results from Central Sumatra^62^  **Early 2015:** Retrospective identification of locally acquired ZIKV identified in Jambi province, Sumatra from blood sample taken early 2015 (reported 15 April 2016)^62,63^  **15 Nov 2015**: case of viral detection a symptomatic case^16^ (Archive Number: 20151115.3792202) | 31 Jan 2016: one case of ZIKV reported^16^ (Archive number 20160203.3990632) | **May 2012**: 52 year old female traveler who returned from Indonesia with fever and rash^64^ (***GeoSentinel case and sentinel marker of disease activity***)*  **Reported in 2015 (travel dates not included):** case of an Australian traveler who appeared to contract ZIKV in Indonesia in whom transmission via a monkey bite was postulated^65^  2 cases imported into Europe, **most recently 24-30 April 2016**^19^  **May 2016**: 1 imported cases to NZ^66^  **1 June 2016:** traveler from Indonesia (east Java Province) with ZIKV diagnosed in Taiwan  **6 June 2016:** traveler from Indonesia entering Taiwan was detected with ZIKV by airport quarantine officers^67^ | Endemic, risk to travelers is low  **30 Sep 2016:** Included in CDC’s Southeast Asia Regional special considerations notice | **5 Feb 2016**: viral circulation 14 April 2016: past transmission, with or without ongoing transmission **7 July to Dec 2016:** Category 2 | **17 June 2016 to 2 Sep 2016**: appeared as sporadic transmission | **27 July 2016 to Dec 2016:** moderate risk |
| Lao PDR | **2015:** Retrospective testing (in 2016) of samples performed by the Institut Pasteur du Laos and demonstrated the presence of ZIKV in 1.3% of specimens tested^16^ (ProMed Archive Number 20160301.4059896) | **4 March 2016:** locally acquired cases reported^16,18^ (Archive Number 20160311.4086075) |  | **30 Sep 2016:** Included in CDC’s Southeast Asia Regional special considerations notice | **4 March 2016:** autochthonous transmission 14 April 2016: past transmission, with or without ongoing transmission **7 July to Dec 2016**: Category 3 | Past transmission | 4 Nov 2016:  classified as low risk based on recent reported transmission |
| Malaysia | **1951-1954:** serological studies suggested human ZIKV exposure^68,69^ – 1st suspected cases outside Africa  **1966:** ZIKV isolated from Aedes aegypti mosquitoes^29^, represented the 1^st^ definitive isolation outside Africa  **1996-1998:** evidence of ZIKV infection in orangutans living in Malaysia and in human blood samples^69,70^ | **3 Sep 2016**: 1^st^ local mosquito-borne transmission confirmed in Sabah (1^st^ imported case was found positive of ZIKV on 31 August)^18^  **7 Sep 2016**: 2^nd^ case reported in woman whose husband commutes daily to Singapore for work^18^ (***example of travelers as “vectors” of disease***)^#^  **11 Sep 2016**: 3rd locally acquired case (25-year-old man who commutes daily to Singapore for work)^18^ (***example of travelers as “vectors” of disease***)^#^  **14 Sep 2016:** 40 suspected cases, 2 further confirmed cases^18^  **29 Sep 2016:** 70 confirmed cases. Testing on samples from 5 ZIKV cases 5indicate the virus contracted locally is from the Asian lineage (2 cases similar to the strain circulating in French Polynesia since 2013, 3 cases are from older Asian lineage previously circulating in Southeast Asia)  **2 Oct 2016**: Locally acquired ZIKV cases also reported in Sabah, Malaysia^18^  **Dec 2016**: confirmed case of ZIKV | **Aug 2014**: 45 yo female traveler who visited Sabah and peninsular Malaysia diagnosed with ZIKV infection after returning to Germany^16,71^ (Archive number 20150517.3367412) (***example of traveler as sentinel***)^  **Aug 28-3 Sep 2016:** Case imported into Europe^19^  **9-17 Oct 2016:** 3 exported cases from Malaysia to Taiwan woman^67^ | Endemic, risk to travelers is low  **30 Sep 2016:** Included in CDC’s Southeast Asia Regional special considerations notice | **5 Feb 2016**: viral circulation 14 April 2016: past transmission, with or without ongoing transmission **7 July:** Category 3  **8 Sep to Dec 2016:** changed from Category 3 to Category 2 | **9 Sep 2016 to Dec 2016:** Appeared as sporadic transmission | **5 Sep 2016**: Changed from low to moderate risk |
| Maldives |  | **June 2016:** reports of suspected locally-acquired cases | **June 2015:** case in 37 yo man who ZIKV imported into Finland (confirmed by PCR of urine)^72^ (***example of traveler as sentinel***)^  **Feb 2016:** importation to Spain by a 53 year old man, plus subsequent report of probable sexual transmission of ZIKV in Spain in this couple^73^ (***example of traveler as sentinel***)^  **22 June 2016:** case imported into Germany^16,19^ (Archive Number: 20161001.4529740} (***GeoSentinel case***)* | Endemic, risk to travelers is low  **30 Sep 2016:** Included in CDC’s Southeast Asia Regional special considerations notice | **5 Feb 2016**: autochthonous transmission  **14 April 2016:** outbreak  **7 July 2016**: Category 3  **29 Sept to Dec 2016:** moved from Category 3 to Category 2 | **21 Jan 2016:** autochthonous in prior 9 months  **From 8 Feb 2016 to Oct 7:** Historical/past transmission  **14^th^ Oct to Dec 2016:** reappeared on list as sporadic transmission | **22 Sep 2016**: changed from low to moderate risk |
| Myanmar |  | **27 Oct 2016:** reported confirmation of the first case of ZIKV infection in Myanmar, subsequently reported to have been transmitted via person-to-person and not via local mosquitoes **(**the husband of the case had a history of travel to countries with known ZIKV transmission)^16^ (Archive Number 20161104.4606432) imported) |  | **30 Sep 2016:** Included in CDC’s Southeast Asia Regional special considerations notice | No risk (not listed) |  | **As at 10 Nov 2016**: No risk (not listed) |
| Pakistan | **1980:** serologic evidence of human ZIKV infection reported (2.3% seroprevalence)^74^ |  |  | Endemic, risk to travelers is low | No risk (not listed) |  | No risk (not listed) |
| Philippines | **1953:** serological study reported suggesting evidence of human ZIKV infection^75^  **2012**: 1^st^ case of ZIKV reported^76,77^ | **March 2016:** WHO timeline lists Philippines as a country with ZIKV^78^  **5 Sep 2016**: 1^st^ locally acquired infection in the Philippines^18^  **21 Sep 2016:** total of 9 locally acquired ZIKV infections in the Philippines. **By 26 Sep**, 12 cases^18^  **17 Oct 2016**: 17 cases from 4 different regions^16,18^ (Archive Number 20161104.4606432)  **4 Nov 2016**: 6 more cases, bringing total cases to 23^18^  **6 Dec** **2016**: total of 39 cases^18^ | **22 Dec 2014:** case imported into Germany (***GeoSentinel case, 1^st^ case since 2012 so sentinel marker of disease activity***)*  **6 March 2016:** ZIKV case confirmed in female traveler after returning to the US (1st ZIKV case detected in Philippines since 2012). Local transmission confirmed either in March^16,18^ (Archive Number 20160311.4086075)  **May-June 2016**: Two case imported to South Korea^16,79^ (Archive number 20160504.4202525)  **31 July-6 Aug 2016:** Case imported into Europe^19^  **18 September 2016**: Case imported into South Korea^16^ | Endemic, risk to travelers is low  **30 Sep 2016:** Included in CDC’s Southeast Asia Regional special considerations notice | **5 Feb 2016**: viral circulation 14 April 2016: past transmission, with or without ongoing transmission **7 July to Dec 2016:** Category 2 | 10 March to 5 Aug 2016: Classified as sporadic transmission12 Aug to 2 Sep 2016: Not mentioned9 Sep 30 to 23 Sep 2016: sporadic transmission **30^th^ Sep to Dec 2016:** changed to increasing or widespread transmission | **28 Sep 2016:** changed from moderate to high risk |
| Singapore |  | **13 May 2016**: 1^st^ imported case of ZIKV in 48 yo male traveler to Sao Paulo, Brazil, but subsequent sequencing of indicate a likely evolution from pre-2007 virus strains already circulating in Southeast Asia since the 1960s and not recently imported from South America^18^  **27 August 2016:** first case of locally-acquired ZIKV in Singapore confirmed^80^. By 28 August: At least 41 cases of ZIKV infection have been reported. By 29 August: 56 cases. By 30 August 2016: > 80 cases. 31 August: 115 cases, one of whom is a pregnant woman^18^  **3 Sep 2016**: Singapore: > 200 locally acquired cases, shown to be "Asian lineage” closer to strains from South East Asia than strain currently causing outbreaks in Americas)^18^  **12 Sep 2016:** Singapore: > 320 cases of non- travel related ZIKV infection including 8 pregnant women and 7 clusters^18^  **29 Sep 2016:** 392 cases (8 active clusters)^18^  **20 Oct 2016:** total of 415 cases reported in 2016^18^  **28 Nov 2016:** 454 cases reported (12 clusters)^18^ | **Aug 2016:** ZIKV case exported to Malaysia^79^  **12 Sep 2016:**   new imported ZIKV case from Singapore reported in Taiwan^18,67^ | **30 Aug 2016:** Level 2 travel warning | **1 Sep 2016:** Added as Category 1 | **9 Sept 2016:** Classified as increasing or widespread transmission | **30 August 2016:** Singapore added to country list, high risk |
| Thailand | **1954:** serological study reported suggesting evidence of human ZIKV infection^81^  **2001–2012:** retrospective seroepidemiological study showing evidence of ZIKV transmission^82^  **2012-2014:** Retrospective identification of 3 locally acquired cases from March 2012 after exportation of infection in 2 travelers, with additional sporadic cases in 2013 (2 cases) and 2014 (2 cases)^71,83,84^ | **Jan 2016:** 1st case of locally-acquired ZIKV for 2016^16,18^ (Archive number 20160217.4026836)  **23 June 2016**: 5 sporadic locally acquired cases^18^  **1 July 2016:** > 90 cases from 10 different provinces reported in first half of 2016 (no evidence of a widespread outbreak)^18^  **16 Sep 2016**: 279 cases (on average, 20 new ZIKV infections reported each week^18^  **30 Sep 2016**: 2 cases of microcephaly (1^st^ cases of microcephaly in Asia)^18^  **16 Nov:** Report of > 680 cases of ZIKV since January 2016 | **Jan 2013:** ZIKV RNA detected in Canadian woman recently returned from southern Thailand^83,85^  (also reported in^16^, Archive Number 20130529.1744108) (***GeoSentinel case and sentinel marker of disease activity***)*  **Nov 2013**: Case reported in German male who developed symptoms 12 days after arrival in Thailand^16,86^ (Archive number 0131227.2139786)  **May 2014**: Importation into Italy (diagnosed retrospectively in March 2016), evidence of sexual transmission of ZIKV on return home^79,87^  **July 2014**: ZIKV infection reported in 41-year old Japanese male traveler to southern Thailand^16,88^ (Archive number 20140823.2716731) (***GeoSentinel case***)  **3 Sep 2014:** ZIKV exported in traveler returning to Germany (***GeoSentinel case***)*  **Oct-Nov 2015:** Retrospective diagnosis (made in March 2016) of ZIKV in semen of French traveler 93 days after returning from Thailand (developed mild illness in early December). Case suggested prolonged potential risk of sexual transmission  **10 Jan 2016:** Confirmation of exported case to Taiwan (detected at the Taipei airport)^16,89^ (Archive number 20160128.3974426)  **21 May 2016**: 2^nd^ case imported into Taiwan^67^  **11-17^h^ Sep 2016:** case imported into Europe^19^  **24 Sep 2016**: Case imported into South Korea^16^  **14 Oct 2016**: case imported from Thailand into Taiwan^67^  **17 Oct 2016**: case imported into Taiwan^67^ | Endemic, risk to travelers is low  **30 Sep 2016:** Included in CDC’s Southeast Asia Regional special considerations notice | **5 Feb 2016**: viral circulation 14 April 2016: past transmission, with or without ongoing transmission **7 July to Dec 2016:** Category 2 | **21 Jan 2016**: autochthonous transmission reported in prior 2 months 21^st^ Jan to 1^st^ April 2016: Sporadic transmissionfollowing recent introduction8 April to 27 May 2016: historical/past transmission3 June 2016: changed to sporadic transmission8 July to Dec 2016: changed to widespread transmission | **Since 25 July 2016:** high risk |
| Timor Leste |  |  | **26 June 2016**: report of probable ZIKV imported case into Germany (patient presented and diagnosed in June although had returned from travel 14-28 April 2016) (***GeoSentinel case and sentinel marker of disease activity***)* | **30 Sep 2016:** Included in CDC’s Southeast Asia Regional special considerations notice | No risk (not listed) |  |  |
| Vietnam | **1954:** serological study reported suggesting evidence of human ZIKV infection^81^ | **5 April 2016**: 2 cases of locally acquired ZIKV confirmed^18^  **6 August 2016**: 3^rd^ locally-acquired ZIKV case confirmed^18^  **9 Oct 2016:** 2 new locally acquired cases (total of 5 in 2016)^18^  **17 Oct 2016:** 1^st^ confirmation of microcephaly and other central nervous system malformations (ZIKV strain is also unknown)  **20 Oct 2016:** 4 further locally acquired cases (total of 9 in 2016). **By 26 Oct 2016**: 11 cases. **By 31 Oct 2016**: 23 cases. **By 3 Nov** 2**016**: 39 cases^16,18^ (Archive Number 20161104.4606432)  **4 Nov 2016:** total of 44 locally acquired cases^18^  **25 Nov** **2016**: >75 cases reported^18^  **13 Dec 2016**: >116 cases reported^18^, including one possible case of microcephaly^16^ (Archive number 20161207.4680914) | **Dec 2015:** exported ZIKV case from Vietnam to Israel by 51 yo male traveler^16,90^ (Archive number 20160317.4102468) (***GeoSentinel case and sentinel marker of disease activity***).* Phylogenetics showed this to be due to the Asian lineage (similar to Pacific outbreak)  **March 2016:** case reported in an Australian traveler (referred to in^90^, documented on Australian Surveillance^91^)  **April 2016:** One imported case confirmed in South Korea in a patient who had spent time in Ho Chi Minh area^16,84^ (Archive number 20160511.4214303)  **17 June 2016**: case exported to Germany^16^ (Archive number 20160707.4331999)  **July-Aug 2016**: 2 further cases imported into South Korea^52,79^  Total of 3 cases have been imported into Europe, most recent **21-27 Aug 2016**^19^  **12 Sep 2016**: imported ZIKV into Japan (11th case of travel-related ZIKV infection for Japan since 2013) ^16^ (Archive number 20160915.4491053) (***GeoSentinel case***)*  **23 Sep 2016:** imported case into Taiwan (eighth imported ZIKV case)^16^ (Archive number 20160922.4506931)  **2 Oct 2016:** case imported into Taiwan^67^ | Endemic, risk to travelers is low  **30 Sep 2016:** Included in CDC’s Southeast Asia Regional special considerations notice newly introduced and spreading widely | **7 April 2016:** past transmission, with or without ongoing transmission  **7 July to Dec 2016:** Category 2 | 24 March to 1^st^ April 2016: listed as sporadic transmission8 April to 29 July 2016: Increasing or widespread5^th^ Aug to 4^th^ Nov 2016: sporadic4 Nov to Dec 2016: changed to widespread | **Since 5 Aug 2016:** moderate risk  **18 Oct 2016:** updated risk classification to high risk based on recent reported transmission |
| Other:  Brunei |  |  |  | **30 Sep 2016:** Included in CDC’s Southeast Asia Regional special considerations notice | No risk (not listed) | Not listed | Not listed |
| Pacific | | | | | | | |
| American Samoa |  | **21 Jan 2016:** reported new autochthonous transmission^17^  **Feb 2016:** Outbreak confirmed, >400 cases suspected  **July – 13 Nov 2016**: further 230 cases, giving total of 944 suspected cases (including 55 confirmed cases) since Jan 1 2016 | **Jan 2016:** American Samoa exported case to New Zealand^  (Note: although American Samoa had suspected cases of ZIKV, this exportation occurred prior to confirmation of outbreak, so example of ***traveler as sentinel***)^66^^  **6 Feb 2016**: child reported as confirmed ZIKV case after return to Australia^18^  **17^th^ Feb 2016:** 2^nd^ imported case to New Zealand  **Feb 2016**: imported case into USA from traveler (visited American Samoa 18 to 25 Jan 2016, diagnosed 29 Feb) (***GeoSentinel case***)* | **1^st^ Feb 2016 to Dec 2016:** Epidemic, Level 2 travel warning | **12 Feb 2016:** autochthonous transmission  **14 April:** outbreak  **7 July to Dec 2016:**  Category 1 | **5 Feb 2016:** Sporadic transmission  **12 Feb** **to Dec 2016:** Increasing or widespread | **Since 25 July 2016:** high risk |
| Cook Islands | **Feb-May 2014**: outbreak in Cook Islands, 932 suspected cases, 52 confirmed^92,93^. First case occurred in traveler from French Polynesia^17^, highlighting potential for ***travelers as “vectors” of disease***^#^ |  | **2010-2014**: 4 cases imported into USA^94^  **Early 2014**: 1 case exported to New Caledonia^95^ (cases occurred simultaneously with the first reports of locally acquired cases, so example of ***traveler as sentinel***)^  **March 2014**: case of ZIKV infection in 65 yo female exported to Australia^16,96^ (Archive number 20140403.2378034), and another 8 cases exported over next few months^97^  **First half of 2014**: 29 exported cases to New Zealand^16,97^ (Archive number 20140910.2762121) and one case to UK ^16^ (Archive number 20160217.4026836)  **July-Dec 2014:** additional 14 exported cases to New Zealand^16,97^ (Archive number 20140910.2762121)  **April 2015**: case imported ZIKV into Australia^98^  **July 2015:** San Diego resident returning from a trip to Cook Islands reported to be diagnosed with ZIKV^99^ | **No mention** | **14 April 2016:** past transmission, with or without ongoing transmission  **7 July to Dec 2016**:  Category 3 | Past transmission |  |
| Fiji | **July – Aug 2015:** evidence of viral transmission, but number of cases uncertain^17,100,101^  **16 Aug 2015**: 2 cases were confirmed to be RT-PCR positive^102^ | **14 Feb 2016**: 4 suspected cases reported all of which had recent traveled to a country affected with ZIKV^102^  **Feb to Aug 2016**: Over 35 confirmed cases reported through late August 2016^16^ (Archive Number 20160311.4086075) | **Aug 2015:** 7 imported cases into Australia^91^ (case occurred simultaneously with the first reports of locally acquired cases, so example of ***traveler as sentinel***)^  **Feb 2016:** Case imported into China^16,79^ (Archive Number 20160301.4059896)  **April 2016**: 3 imported cases to New Zealand^66^  **May 2016**: 5 imported cases to New Zealand^66^  **July 2016:** 1 imported cases to New Zealand^66^  2 cases imported into Europe, most recently **24-30 July 2016**^19^ | **4^th^ April 2016:** Epidemic, Level 2 travel warning (remains Level 2 even though no transmission has been documented last 3 months or more) | **5 Feb 2016**: autochthonous transmission  **April 14 2016:** outbreak  **7 July to Dec 2016**: Category 1 | **Affected in 2015**  **21 Jan to 4 March 2016**: autochthonous in prior 9 months 10 March to 1 April 2016: changed to sporadic transmission **8 April to Dec 2016**: Increasing or widespread | 4 Nov 2016:  [updated](http://travelhealthpro.org.uk/news/4/zika-virus--update-and-advice-for-travellers-including-pregnant-women-and-those-planning-pregnancy) to high risk based on recent reported transmission |
| French Polynesia | **2011-2013**: 0.8% of blood donors had antibodies to ZIKV^103^  **Nov 2013 to April 2014:** outbreak occurred with 8750 suspected ZIKV cases, 383 PCR confirmed cases, and total estimate of 32 000 clinical cases occurring (11.5% of the total population). Origin of outbreak unknown. Retrospective identification of 8 cases of birth defects and 42 cases of GBS. Exportation to New Caledonia, Cook Islands and Easter Island^16,93,104-107^, and confirmation of transmission via semen^108^. Highlights potential for ***travelers as “vectors” of disease***^#^ |  | **Nov 2013**: exported to France by 51 yo female traveler^109^ (example of ***traveler as sentinel***)^  **Nov 2013**: case exported to New Caledonia (subsequently leading to outbreak there in Jan 2014, total of 32 importations in 2014)^95^  **Dec 2013-Jan 2014:** 2 cases of ZIKV exported to Japan^16,110^ (Archive Number: 20131219.2126046)  **Dec 2013:** 31 yo female exported to Norway from Tahiti^16,111^ (Archive number 20140303.2309965) (***GeoSentinel case***)*  **Dec 2013**: case in 48 yo male imported into the US^112^  **3 Jan 2014:** imported case into France (***GeoSentinel case***)*  **14 Jan 2014:** imported case to US (probably included in^94^ (***GeoSentinel case***)*  **Jan 2014:** 2 cases imported into Italy^113^  **2010-2014:** 6 cases imported into USA^94,114,115^  **25 Sep 2014**: 27 yo Belgian traveler exported ZIKV^16^ (Archive Number 20140925.2804821) | (No mention) | **12 Feb 2016:** outbreak terminated  **14 April 2016**: country with past transmission, with or without ongoing transmission  **7 July to Dec 2016:** Category 3 |  |  |
| Marshall Islands |  | **Feb 16 2016:** first report of presumed locally acquired ZIKV infection  **3 March 2016:** state of emergency declared following confirmation of 1^st^ ZIKV case^18^  **19 April 2016:** total of 34 locally acquired cases of ZIKV infection reported^102^  **6 May 2016:** Case of microcephaly suspected to be associated with ZIKV infection reported | **Jan 2016:** Ministry of Health was first alerted of an outbreak in late Jan via report of a traveler who was ill in November after a 3 week stay in Majuro, who returned to Australia and was **diagnosed on 22 Nov 2015**^116^ (example of ***traveler as sentinel***)^ | **23 Feb 2016 to Dec 2016**: Epidemic, Level 2 travel warning | **26 Feb 2016**: autochthonous transmission  **7 July 2016 to Dec 2016:** Category 1 | **Feb 19 to 10 March 2016:** sporadic transmission  **24 March to 1 July 2016:** Increasing or widespread  **From 8 July to Dec 2016:** not listed | Low risk |
| Micronesia | **April 2007:** Island of Yap, 1^st^ large outbreak of ZIKV with 49 confirmed and 59 probable cases, 77 suspected; estimated that 73% of the population was infected. No deaths, hospitalizations, or neurological complications reported. Subsequently found to be due to strain from Southeast Asia^117,118^. Notable outbreak as 1^st^ time that ZIKV was detected outside of Africa and Asia | **Jan 2016:** locally acquired case of ZIKV in a person in Kosrae State who had not traveled abroad  **22 May 2016**: total of 90 cases, including 11 confirmed cases^102^  Ongoing outbreak since, now past its peak but mid-Sep to mid-October >20 cases reported  **1 Nov 2016:** emergency declaration for mosquito borne diseases declared (total of 157 cases reported, 23 confirmed / 21 probable)  **30 Nov 2016: total of** 238 cases reported | **2007 outbreak:** One case from Yap exported to USA^117^ and 2 possible cases exported to Guam^16^(Archive number 20070627.2065 and 20070703.2115)  **20 Sep 2016:** ZIKV exported into the USA in a pregnant traveler (***GeoSentinel case***)* | **1 April 2016 to Dec 2016:** Epidemic, Level 2 travel warning (Kosrae) | **12 Feb 2016:** outbreak terminated  **14 April 2016:** past transmission, with or without ongoing transmission  **7 July to Dec 2016:** Category 1 | **1 April to 19 April 2016:** sporadic transmission (Kosrae)  **13 May to Dec 2016:** Increasing or widespread (Kosrae) | **25 July 2016 to Dec 2016:** High risk |
| New Caledonia | **January 2014 to Oct 2014:** outbreak with 1385 confirmed cases (0.8% of the population), including 35 imported cases (Note: outbreak was preceded by importation of cases from French Polynesia), with 32 case importations from French Polynesia, 2 from Vanuatu and 1 case from Cook Islands)^92,93,95,101^, and possibly exported to Vanuatu^119^. Highlights potential for ***travelers as “vectors” of disease***^#^  **2015:** ZIKV still circulating, 137 cases by Aug | **4 March 2016**: reported cases of locally-transmitted ZIKV^16,18^ (Archive Number 20160311.4086075)  **By mid-June 2016:** total of 7 locally acquired, confirmed cases of ZIKV infection reported since 2016^18^ |  | **7 Feb 2014:** Level 1 warning removed 5 Aug 2014  **9 March 2016 to Dec 2016:** Epidemic, Level 2 travel warning | **5 Feb 2016:** Past transmission/outbreak terminated  **14 April 2016:** past transmission, with or without ongoing transmission  **7 Jul 2016:** Category 3  **6 Oct to Dec 2016**: changed to Category 2 | **21^st^ Jan 2016** (1^st^ ECDC Zika update): autochthonous cases of ZIKV infection have been reported in 2015 (January to early August)  **4 March 2016 to 3 June**: sporadic transmission  (Not listed since) |  |
| Palau |  | **7 Nov 2016**: 1^st^ locally acquired, confirmed case of ZIKV infection reported | **14 Nov 2016:** ZIKV diagnosed in a patient who returned home to the Netherlands (illness onset 8 Oct 2016) (***GeoSentinel case, coincident with first reported local case***)* | **16 Nov 2016**: Local transmission, Level 2 travel warning | **10 Nov 2016:** added to category 1 | **10 Nov 2016:** added as Sporadic transmission | **8 Nov 2016:** Added Palau as high risk |
| Papua New Guinea (PNG) | **May 2015:** one case of locally acquired ZIKV suspected  **Dec 2015**: two cases of locally acquired ZIKV | **Feb 16:** 3 further cases of locally acquired ZIKV reported | **10-16 Jan 2016:** 1 case imported into Europe^19^  **1 April 2016:** 2 exported cases from PNG to New Zealand reported^66^ | **29 April 2016 to Dec 2016:** Epidemic, Level 2 travel warning | **17 March 2016**: autochthonous transmission  **14 April 2016:** past transmission, with or without ongoing transmission  **7 July to Dec 2016:** Category 3 | **1 April 2016 to 10 June:** sporadic transmission  **Since 17 June 2016**: past transmission | **18 October 2016:** updated from low to moderate risk |
| Samoa | **Early 2015:** sporadic cases of locally acquired ZIKV reported^16^ (Archive Number: 20151205.3842908)  **13 Sep 2015**: ZIKV identified in one sample sent for confirmatory testing^102^  **15 Nov 2015**: total of 3 ZIKV cases identified (RT-PCR positive)^102^ | **Jan -Feb 2016**: additional cases of locally acquired ZIKV reported  **May 2016**: >160 cases reported in the outbreak since Aug 2015, including 24 confirmed cases^102^ | **Dec 2015 – Jan 2016**: Exported 4 cases from Samoa to NZ^66^  **10-16 Jan 2016**: ECDC reports 1 case imported into Europe^19^  **3-10 Feb 2016**: 9 additional cases imported into NZ^66^  **11-17 Feb 2016**: 4 additional cases imported into NZ^66^  **18-24 Feb 2016**: 1 additional case imported into NZ^66^  **Feb 2016**: 3 cases imported into China (plus a 4^th^ case who had been to both Fiji and Samoa)^16,120^ (Archive Number 20160301.4059896)  **25 Feb – 2 Mar 2016**: 2 additional cases imported into NZ^66^  **By Nov 2016:** Total of 5 imported cases into Australia^91^ | **22 Jan 2016 to Dec 2016**: Epidemic, Level 2 travel warning | **5 Feb 2016**: autochthonous transmission  **April 14 2016:** outbreak  **7 July to Dec 2016**: Category 1 | **21 Jan 2016:** autochthonous in prior 9 months  **4 Feb 2016:** sporadic transmission following recent introduction  **15^th^ April to 14^th^ Oct 2016:** Increasing or widespread  **Since 21^st^ Oct 2016**: not listed | High risk |
| Solomon Islands | **Feb 2015:** locally acquired cases of ZIKV reported^17^  **24 May 2015**: total of 310 cases since Feb 2015^102^ | **Since Jan 2016:** low-level ongoing transmission suspected  **6 April 2016:** 240 Zika-like illness cases since Feb 2015^102^  **17 August to 24 Oct 2016:** ongoing transmission reported^17^ | **10-16 Jan 2016**: ECDC reports 1 case imported into Europe^19^  **Feb 2016**: case imported into Australia^100,121^  **12 Oct 2016**: Australia reported ZIKV case infection in a returning traveler from the Solomon Islands^19^ | (No mention) | **5 Feb 2016**: autochthonous transmission 14 April 2016: past transmission, with or without ongoing transmission7 July to Dec 2016: Category 3 | Affected in 2015  **21 Jan – 22 April 2016:** autochthonous in prior 9 months  **April to Nov 2016:** not listed  **4 Nov** **2016 to 18^th^ Nov** sporadic transmission  **18^th^ Nov to Dec 2016**: not listed | 18 October 2016: Updated Solomon Islands from low to moderate risk20 Oct 2016: Changed Solomon Islands from moderate to high risk |
| Tonga |  | **Jan 2016:** outbreak reported, with >860 suspected cases  **27 March 2016**: 2420 cases since 1 January 2016^102^ | **10-16 Jan 2016:** 1 case imported into Europe^19^ (case occurred simultaneously with 1^st^ reports of locally acquired cases, so example of ***traveler as sentinel***)^  **1 Jan-3 Feb 2016:** 5 exported cases from Tonga to NZ^66^  **3-17 Feb:** 20 additional cases imported to New Zealand by travelers^66^  **18 Feb -2 Mar 2016:** 18 additional cases imported into New Zealand^66^  **Feb 2016:** Case exported to Australia by female traveler^122^ (Note: a total of 11 confirmed imported cases by Nov 2016)^91^  **7 March 2016**: 2 cases diagnosed in travelers returning to USA (***GeoSentinel cases***)*  **3 -30 March 2016**: 3 additional cases imported into New Zealand^66^  **April 2016**: 1 imported case to New Zealand^66^  **9 May 2016**: imported case to Australia^16^ (Archive number 20160524.4240474) | **3 Feb 2016 to Dec 2016:** Epidemic, Level 2 travel warning | **5 Feb 2016**: autochthonous transmission  **April 14 2016:** outbreak  **7 July to Dec 2016**: Category 1 | **21 Jan 2016:** new autochthonous transmission  **5 Feb 2016:** appeared as increasing or widespread transmission | **16 September 2016 to Dec 2016:** moderate risk |
| Vanuatu | **Feb –May 2015:** locally acquired cases of ZIKV reported, possibly exported from New Caledonia^17,119^ | **Jan - April 2016**: Low-level ongoing transmission suspected  **26 April 2016**: first case confirmed using PCR^102^ | **Early 2014**: 2 cases exported to New Caledonia^95^  **First half of 2014**: 1 case exported New Zealand^97^  **Jan to March 2015**: 6 cases exported to New Caledonia^102^  **24-30 Jan 2016**: ECDC reports 1 case imported into Europe^19^ (cases occurred simultaneously with 1^st^ reports of locally acquired cases in 2016, showing role ***travelers play as sentinels***)^ | Endemic, risk to travelers is low | **5 Feb 2016**: autochthonous transmission  **14 April 2016:** past transmission, with or without ongoing transmission  **7 July to Dec 2016**:  Category 3 | **Affected in 2015**  **21 Jan 2016 – 15 April:** autochthonous in prior 9 months  Not listed since then | Low risk |
| Other:  Kiribati |  |  | **30 April 2015:** ZIKV case imported into New Zealand (***GeoSentinel case and sentinel marker of disease activity***)*  **July 2015:** San Diego resident returning from a trip to Kiribati was reported to have been diagnosed with ZIKV^99^ | (No mention) | No risk (not listed) |  | Kiribati: no risk (not listed) |

ZIKV: Zika virus; yo: years old

*GeoSentinel case

^Example of non-GeoSentinel traveler as sentinel

^#^Example of traveler as “vector” of disease

Where specific references are absent, the epidemiological data have been gathered from the following sources: journal publications^15,63,79,84,101,123,124^, International SOS reports^125^, WHO timeline^78^, WHO situation reports^4^, WHO Western Pacific Region reports^102^, ECDC situation reports^5^ and Rapid Risk Assessments^17^, ProMed reports^16^ or online news reports.

**References**

1. CDC: Zika Virus Disease and Zika Virus Infection 2016 Case Definition, CSTE Position Statement(s) (Approved June 2016). (Accessed 10 Nov 2016, 2016, at https://wwwn.cdc.gov/nndss/conditions/zika/case-definition/2016/06/.)

2. DH Hamer, Barbre KA, Chen LH, et al. Travel-Associated Zika Virus Disease Acquired in the Americas Through February 2016: A GeoSentinel Analysis. Ann Intern Med 2016;Nov 22. doi:10.7326/M16-1842.

3. CDC: Zika travel information. (Accessed 15 Dec, 2016, at <http://wwwnc.cdc.gov/travel/page/zika-travel-information.>)

4. WHO: Zika virus situation reports. (Accessed 13 Dec, 2016, at <http://www.who.int/emergencies/zika-virus/situation-report/en/.>)

5. ECDC: Zika virus situation reports. (Accessed 13 Dec, 2016, at <http://ecdc.europa.eu/en/healthtopics/zika_virus_infection/zika-outbreak/Pages/epidemiological-situation.aspx.>)

6. NaTHNaC: Zika virus: country specific risk. (Accessed 26 Nov, 2016, at https://[www.gov.uk/guidance/zika-virus-country-specific-risk#atoz.](http://www.gov.uk/guidance/zika-virus-country-specific-risk#atoz.))

7. P Bres. [Recent data from serological surveys on the prevalence of arbovirus infections in Africa, with special reference to yellow fever]. Bull World Health Organ 1970;43:223-67.

8. WHO: Countries and territories showing historical transmission of Zika virus, 1947-2016. (Accessed 8 Nov, 2016, at <http://www.who.int/emergencies/zika-virus/zika-historical-distribution.pdf?ua=1.>)

9. G Grard, Caron M, Mombo IM, et al. Zika virus in Gabon (Central Africa)--2007: a new threat from Aedes albopictus? PLoS Negl Trop Dis 2014;8:e2681.

10. AD Haddow, Schuh AJ, Yasuda CY, et al. Genetic characterization of Zika virus strains: geographic expansion of the Asian lineage. PLoS Negl Trop Dis 2012;6:e1477.

11. JJ Salaun, Brottes H. [Arbovirus in Cameroon: serologic investigation]. Bull World Health Organ 1967;37:343-61.

12. JJ Salaun, Rickenbach A, Bres P, et al. [Arboviruses isolated from mosquitoes in Cameroon]. Bull World Health Organ 1969;41:233-41.

13. EB Fokam, Levai LD, Guzman H, et al. Silent circulation of arboviruses in Cameroon. East Afr Med J 2010;87:262-8.

14. M Van Esbroeck, Meersman K, Michiels J, Arien KK, Van den Bossche D. Letter to the editor: Specificity of Zika virus ELISA: interference with malaria. Euro Surveill 2016;21.

15. MK Kindhauser, Allen T, Frank V, Santhana RS, Dye C. Zika: the origin and spread of a mosquito-borne virus. Bull World Health Organ 2016;94:675-86C.

16. ProMed mail reports. (Accessed 20 Oct to 2 Dec, 2016, at <http://www.promedmail.org/.>)

17. ECDC: Zika epidemics 2014 onwards and Rapid Risk Assessments. (Accessed 22 Nov, 2016, at <http://ecdc.europa.eu/en/healthtopics/zika_virus_infection/zika-outbreak/Pages/zika-outbreak.aspx.>)

18. International SOS (iSOS): Zika News. (Accessed 13 Dec, 2016, at https://pandemic.internationalsos.com/zika/zika-news.)

19. ECDC: Place of infection of imported case to the EU (Accessed 20 Nov, 2016, at <http://ecdc.europa.eu/en/publications/surveillance_reports/Communicable-Disease-Threats-Report/Documents/Place%20of%20infection%20of%20imported%20cases%20of%20Zika%20virus%20disease.PNG.>)

20. C Chippaux-Hyppolite. [Immunologic investigation on the frequency of arbovirus in man in the Central African Republic. Preliminary note]. Bull Soc Pathol Exot Filiales 1965;58:812-20.

21. JF Saluzzo, Gonzalez JP, Herve JP, Georges AJ. [Serological survey for the prevalence of certain arboviruses in the human population of the south-east area of Central African Republic (author's transl)]. Bull Soc Pathol Exot Filiales 1981;74:490-9.

22. N Berthet, Nakoune E, Kamgang B, et al. Molecular characterization of three Zika flaviviruses obtained from sylvatic mosquitoes in the Central African Republic. Vector Borne Zoonotic Dis 2014;14:862-5.

23. Y Robin, Bres P, Lartigue JJ, et al. [Arboviruses in Ivory Coast. Serologic survey in the human population]. Bull Soc Pathol Exot Filiales 1968;61:833-45.

24. C Akoua-Koffi, Diarrassouba S, Benie VB, et al. [Investigation surrounding a fatal case of yellow fever in Cote d'Ivoire in 1999]. Bull Soc Pathol Exot 2001;94:227-30.

25. KC Smithburn, Taylor RM, Rizk F, Kader A. Immunity to certain arthropod-borne viruses among indigenous residents of Egypt. Am J Trop Med Hyg 1954;3:9-18.

26. C Jan, Languillat G, Renaudet J, Robin Y. [A serological survey of arboviruses in Gabon]. Bull Soc Pathol Exot Filiales 1978;71:140-6.

27. JF Saluzzo, Ivanoff B, Languillat G, Georges AJ. [Serological survey for arbovirus antibodies in the human and simian populations of the South-East of Gabon (author's transl)]. Bull Soc Pathol Exot Filiales 1982;75:262-6.

28. MR Pinto. Survey for antibodies to arboviruses in the sera of children in Portuguese Guinea. Bull World Health Organ 1967;37:101-8.

29. NJ Marchette, Garcia R, Rudnick A. Isolation of Zika virus from Aedes aegypti mosquitoes in Malaysia. Am J Trop Med Hyg 1969;18:411-5.

30. FN Macnamara. Zika virus: a report on three cases of human infection during an epidemic of jaundice in Nigeria. Trans R Soc Trop Med Hyg 1954;48:139-45.

31. DL Moore, Causey OR, Carey DE, et al. Arthropod-borne viral infections of man in Nigeria, 1964-1970. Ann Trop Med Parasitol 1975;69:49-64.

32. AH Fagbami. Zika virus infections in Nigeria: virological and seroepidemiological investigations in Oyo State. J Hyg (Lond) 1979;83:213-9.

33. A Fagbami. Epidemiological investigations on arbovirus infections at Igbo-Ora, Nigeria. Trop Geogr Med 1977;29:187-91.

34. EO Adekolu-John, Fagbami AH. Arthropod-borne virus antibodies in sera of residents of Kainji Lake Basin, Nigeria 1980. Trans R Soc Trop Med Hyg 1983;77:149-51.

35. J Renaudet, Jan C, Ridet J, Adam C, Robin Y. [A serological survey of arboviruses in the human population of Senegal]. Bull Soc Pathol Exot Filiales 1978;71:131-40.

36. E Monlun, Zeller H, Le Guenno B, et al. [Surveillance of the circulation of arbovirus of medical interest in the region of eastern Senegal]. Bull Soc Pathol Exot 1993;86:21-8.

37. A Sow, Loucoubar C, Diallo D, et al. Concurrent malaria and arbovirus infections in Kedougou, southeastern Senegal. Malar J 2016;15:47.

38. D Diallo, Sall AA, Diagne CT, et al. Zika virus emergence in mosquitoes in southeastern Senegal, 2011. PLoS One 2014;9:e109442.

39. BD Foy, Kobylinski KC, Chilson Foy JL, et al. Probable non-vector-borne transmission of Zika virus, Colorado, USA. Emerg Infect Dis 2011;17:880-2.

40. Y Robin, Mouchet J. [Serological and entomological study on yellow fever in Sierra Leone]. Bull Soc Pathol Exot Filiales 1975;68:249-58.

41. KC Smithburn. Neutralizing antibodies against certain recently isolated viruses in the sera of human beings residing in East Africa. J Immunol 1952;69:223-34.

42. GW Dick, Kitchen SF, Haddow AJ. Zika virus. I. Isolations and serological specificity. Trans R Soc Trop Med Hyg 1952;46:509-20.

43. GW Dick. Epidemiological notes on some viruses isolated in Uganda; Yellow fever, Rift Valley fever, Bwamba fever, West Nile, Mengo, Semliki forest, Bunyamwera, Ntaya, Uganda S and Zika viruses. Trans R Soc Trop Med Hyg 1953;47:13-48.

44. MP Weinbren, Williams MC. Zika virus: further isolations in the Zika area, and some studies on the strains isolated. Trans R Soc Trop Med Hyg 1958;52:263-8.

45. AJ Haddow, Williams MC, Woodall JP, Simpson DI, Goma LK. Twelve Isolations of Zika Virus from Aedes (Stegomyia) Africanus (Theobald) Taken in and above a Uganda Forest. Bull World Health Organ 1964;31:57-69.

46. DI Simpson. Zika Virus Infection in Man. Trans R Soc Trop Med Hyg 1964;58:335-8.

47. BE Henderson, Metselaar D, Cahill K, Timms GL, Tukei PM, Williams MC. Yellow fever immunity surveys in northern Uganda and Kenya and eastern Somalia, 1966-67. Bull World Health Organ 1968;38:229-37.

48. AW McCrae, Kirya BG. Yellow fever and Zika virus epizootics and enzootics in Uganda. Trans R Soc Trop Med Hyg 1982;76:552-62.

49. F Rodhain, Gonzalez JP, Mercier E, Helynck B, Larouze B, Hannoun C. Arbovirus infections and viral haemorrhagic fevers in Uganda: a serological survey in Karamoja district, 1984. Trans R Soc Trop Med Hyg 1989;83:851-4.

50. RH Kokernot, Casaca VM, Weinbren MP, McIntosh BM. Survey for antibodies against arthropod-borne viruses in the sera of indigenous residents of Angola. Trans R Soc Trop Med Hyg 1965;59:563-70.

51. AR Filipe, De Carvalho RG, Relvas A, Casaca V. Arbovirus studies in Angola: Serological survey for antibodies to arboviruses. Am J Trop Med Hyg 1975;24:516-20.

52. C Serie, Casals J, Panthier R, Bres P, Williams MC. [Studies on yellow fever in Ethiopia. 2. Serological study of the human population]. Bull World Health Organ 1968;38:843-54.

53. A Geser, Henderson BE, Christensen S. A multipurpose serological survey in Kenya. 2. Results of arbovirus serological tests. Bull World Health Organ 1970;43:539-52.

54. RH Kokernot, Smithburn KC, Gandara AF, McIntosh BM, Heymann CS. [Neutralization tests with sera from individuals residing in Mozambique against specific viruses isolated in Africa, transmitted by arthropods]. An Inst Med Trop (Lisb) 1960;17:201-30.

55. ES Gudo, Falk KI, Ali S, Muianga AF, Monteiro V, Cliff J. A Historic Report of Zika in Mozambique: Implications for Assessing Current Risk. PLoS Negl Trop Dis 2016;10:e0005052.

56. OA Babaniyi, Mwaba P, Mulenga D, et al. Risk assessment for yellow Fever in Western and north-Western provinces of zambia. J Glob Infect Dis 2015;7:11-7.

57. V Heang, Yasuda CY, Sovann L, et al. Zika virus infection, Cambodia, 2010. Emerg Infect Dis 2012;18:349-51.

58. V Duong, Ong S, Leang R, et al. Low Circulation of Zika Virus, Cambodia, 2007-2016. Emerg Infect Dis 2017;23.

59. KC Smithburn, Kerr JA, Gatne PB. Neutralizing antibodies against certain viruses in the sera of residents of India. J Immunol 1954;72:248-57.

60. JG Olson, Ksiazek TG, Suhandiman, Triwibowo. Zika virus, a cause of fever in Central Java, Indonesia. Trans R Soc Trop Med Hyg 1981;75:389-93.

61. JG Olson, Ksiazek TG, Gubler DJ, et al. A survey for arboviral antibodies in sera of humans and animals in Lombok, Republic of Indonesia. Ann Trop Med Parasitol 1983;77:131-7.

62. A Perkasa, Yudhaputri F, Haryanto S, et al. Isolation of Zika Virus from Febrile Patient, Indonesia. Emerg Infect Dis 2016;22:924-5.

63. N Wikan, Smith DR. Zika virus: history of a newly emerging arbovirus. Lancet Infect Dis 2016;16:e119-26.

64. JC Kwong, Druce JD, Leder K. Zika virus infection acquired during brief travel to Indonesia. Am J Trop Med Hyg 2013;89:516-7.

65. GH Leung, Baird RW, Druce J, Anstey NM. Zika Virus Infection in Australia Following a Monkey Bite in Indonesia. Southeast Asian J Trop Med Public Health 2015;46:460-4.

66. Public Health Surveillance New Zealand: Zika Virus Infection Report. (Accessed 15 Nov, 2016, at https://surv.esr.cri.nz/surveillance/WeeklyZikaFever.php.)

67. Centres for Disease Control Taiwan (Accessed 30 Nov, 2016, at [www.cdc.gov.tw](http://www.cdc.gov.tw) english.)

68. KC Smithburn. Neutralizing antibodies against arthropod-borne viruses in the sera of long-time residents of Malaya and Borneo. Am J Hyg 1954;59:157-63.

69. ND Wolfe, Kilbourn AM, Karesh WB, et al. Sylvatic transmission of arboviruses among Bornean orangutans. Am J Trop Med Hyg 2001;64:310-6.

70. AM Kilbourn, Karesh WB, Wolfe ND, Bosi EJ, Cook RA, Andau M. Health evaluation of free-ranging and semi-captive orangutans (Pongo pygmaeus pygmaeus) in Sabah, Malaysia. J Wildl Dis 2003;39:73-83.

71. D Tappe, Nachtigall S, Kapaun A, Schnitzler P, Gunther S, Schmidt-Chanasit J. Acute Zika virus infection after travel to Malaysian Borneo, September 2014. Emerg Infect Dis 2015;21:911-3.

72. EM Korhonen, Huhtamo E, Smura T, Kallio-Kokko H, Raassina M, Vapalahti O. Zika virus infection in a traveller returning from the Maldives, June 2015. Euro Surveill 2016;21.

73. M Arsuaga, Bujalance SG, Diaz-Menendez M, Vazquez A, Arribas JR. Probable sexual transmission of Zika virus from a vasectomised man. Lancet Infect Dis 2016;16:1107.

74. MA Darwish, Hoogstraal H, Roberts TJ, Ahmed IP, Omar F. A sero-epidemiological survey for certain arboviruses (Togaviridae) in Pakistan. Trans R Soc Trop Med Hyg 1983;77:442-5.

75. WM Hammon, Schrack WD, Jr., Sather GE. Serological survey for a arthropod-borne virus infections in the Philippines. Am J Trop Med Hyg 1958;7:323-8.

76. MT Alera, Hermann L, Tac-An IA, et al. Zika virus infection, Philippines, 2012. Emerg Infect Dis 2015;21:722-4.

77. DW Ellison, Ladner JT, Buathong R, et al. Complete Genome Sequences of Zika Virus Strains Isolated from the Blood of Patients in Thailand in 2014 and the Philippines in 2012. Genome Announc 2016;4.

78. WHO Zika virus timeline: The origin and spread of a mosquito-borne virus (Accessed 20 Nov, 2016, at <http://www.who.int/bulletin/online_first/16-171082/en/> and <http://www.who.int/emergencies/zika-virus/history/en/.>)

79. D Veasna, Dussart P, Buchy P. Zika virus in Asia. Int J Infect Dis 2016;Dec 6. pii:S1201-9712(16)31640-X.

80. D Fisher, Cutter J. The inevitable colonisation of Singapore by Zika virus. BMC Med 2016;14:188.

81. WL Pond. Arthropod-Borne Virus Antibodies in Sera from Residents of South-East Asia. Trans R Soc Trop Med Hyg 1963;57:364-71.

82. N Wikan, Suputtamongkol Y, Yoksan S, Smith DR, Auewarakul P. Immunological evidence of Zika virus transmission in Thailand. Asian Pac J Trop Med 2016;9:141-4.

83. R Buathong, Hermann L, Thaisomboonsuk B, et al. Detection of Zika Virus Infection in Thailand, 2012-2014. Am J Trop Med Hyg 2015;93:380-3.

84. V Wiwanitkit. The current status of Zika virus in Southeast Asia. Epidemiol Health 2016;38:e2016026.

85. K Fonseca, Meatherall B, Zarra D, et al. First case of Zika virus infection in a returning Canadian traveler. Am J Trop Med Hyg 2014;91:1035-8.

86. D Tappe, Rissland J, Gabriel M, et al. First case of laboratory-confirmed Zika virus infection imported into Europe, November 2013. Euro Surveill 2014;19.

87. G Venturi, Zammarchi L, Fortuna C, et al. An autochthonous case of Zika due to possible sexual transmission, Florence, Italy, 2014. Euro Surveill 2016;21.

88. K Shinohara, Kutsuna S, Takasaki T, et al. Zika fever imported from Thailand to Japan, and diagnosed by PCR in the urines. J Travel Med 2016;23.

89. AS Huang, Shu PY, Yang CH. A new reportable disease is born: Taiwan Centers for Disease Control's response to emerging Zika virus infection. J Formos Med Assoc 2016;115:223-5.

90. E Meltzer, Lustig Y, Leshem E, et al. Zika Virus Disease in Traveler Returning from Vietnam to Israel. Emerg Infect Dis 2016;22:1521-2.

91. Australian Department of Health: Zika virus - notifications of Zika virus infection. (Accessed 28 Nov, 2016, at <http://www.health.gov.au/internet/main/publishing.nsf/Content/ohp-zika-notifications.htm.>)

92. A Roth, Mercier A, Lepers C, et al. Concurrent outbreaks of dengue, chikungunya and Zika virus infections - an unprecedented epidemic wave of mosquito-borne viruses in the Pacific 2012-2014. Euro Surveill 2014;19.

93. VM Cao-Lormeau, Musso D. Emerging arboviruses in the Pacific. Lancet 2014;384:1571-2.

94. MJ Hennessey, Fischer M, Panella AJ, et al. Zika Virus Disease in Travelers Returning to the United States, 2010-2014. Am J Trop Med Hyg 2016;95:212-5.

95. M Dupont-Rouzeyrol, O'Connor O, Calvez E, et al. Co-infection with Zika and dengue viruses in 2 patients, New Caledonia, 2014. Emerg Infect Dis 2015;21:381-2.

96. AT Pyke, Daly MT, Cameron JN, et al. Imported zika virus infection from the cook islands into australia, 2014. PLoS Curr 2014;6.

97. JG Derraik, Slaney D. Notes on Zika virus--an emerging pathogen now present in the South Pacific. Aust N Z J Public Health 2015;39:5-7.

98. N Macesic, Abbott IJ, Johnson DF. PHOTO QUIZ. Fever and Rash in a Husband and Wife Returning From the Cook Islands. Clin Infect Dis 2015;61:1445, 85-6.

99. Frequently Asked Questions – Zika virus and Mosquitoes San Diego County – October 2016. (Accessed 29 Nov, 2016, at <http://www.sandiegocounty.gov/content/dam/sdc/hhsa/programs/phs/documents/Zika_FAQs.pdf.>)

100. KA Taylor, Paterson BJ. Zika virus from a Pacific perspective: What are the risks to Australians? Travel Med Infect Dis 2016;14:159-61.

101. D Musso, Gubler DJ. Zika Virus. Clin Microbiol Rev 2016;29:487-524.

102. WHO, Western Pacific Region, Disease Surveillance (Zika virus). (Accessed 30 Noc, 2016, at <http://www.wpro.who.int/southpacific/programmes/communicable_diseases/disease_surveillance_response/page/en/index2.html.>)

103. M Aubry, Finke J, Teissier A, et al. Seroprevalence of arboviruses among blood donors in French Polynesia, 2011-2013. Int J Infect Dis 2015;41:11-2.

104. E Oehler, Watrin L, Larre P, et al. Zika virus infection complicated by Guillain-Barre syndrome--case report, French Polynesia, December 2013. Euro Surveill 2014;19.

105. M Besnard, Lastere S, Teissier A, Cao-Lormeau V, Musso D. Evidence of perinatal transmission of Zika virus, French Polynesia, December 2013 and February 2014. Euro Surveill 2014;19.

106. JM Jouannic, Friszer S, Leparc-Goffart I, Garel C, Eyrolle-Guignot D. Zika virus infection in French Polynesia. Lancet 2016;387:1051-2.

107. S Cauchemez, Besnard M, Bompard P, et al. Association between Zika virus and microcephaly in French Polynesia, 2013-15: a retrospective study. Lancet 2016;387:2125-32.

108. D Musso, Roche C, Robin E, Nhan T, Teissier A, Cao-Lormeau VM. Potential sexual transmission of Zika virus. Emerg Infect Dis 2015;21:359-61.

109. C Baronti, Piorkowski G, Charrel RN, Boubis L, Leparc-Goffart I, de Lamballerie X. Complete coding sequence of zika virus from a French polynesia outbreak in 2013. Genome Announc 2014;2.

110. S Kutsuna, Kato Y, Takasaki T, et al. Two cases of Zika fever imported from French Polynesia to Japan, December 2013 to January 2014 [corrected]. Euro Surveill 2014;19.

111. T Waehre, Maagard A, Tappe D, Cadar D, Schmidt-Chanasit J. Zika virus infection after travel to Tahiti, December 2013. Emerg Infect Dis 2014;20:1412-4.

112. DJ Summers, Acosta RW, Acosta AM. Zika Virus in an American Recreational Traveler. J Travel Med 2015;22:338-40.

113. L Zammarchi, Stella G, Mantella A, et al. Zika virus infections imported to Italy: clinical, immunological and virological findings, and public health implications. J Clin Virol 2015;63:32-5.

114. KB Brust, Prince WS, Fader RC. Trouble in paradise. IDCases 2014;1:95-6.

115. M McCarthy. First US case of Zika virus infection is identified in Texas. BMJ 2016;352:i212.

116. Zika in Republic of the Marshall Islands, 2015-2016 (Report Date: April 19th, 2016). (Accessed 28 Nov, 2016, at <http://reliefweb.int/report/marshall-islands/zika-republic-marshall-islands-2015-2016-report-date-april-19th-2016.>)

117. MR Duffy, Chen TH, Hancock WT, et al. Zika virus outbreak on Yap Island, Federated States of Micronesia. N Engl J Med 2009;360:2536-43.

118. RS Lanciotti, Kosoy OL, Laven JJ, et al. Genetic and serologic properties of Zika virus associated with an epidemic, Yap State, Micronesia, 2007. Emerg Infect Dis 2008;14:1232-9.

119. D Musso. Zika Virus Transmission from French Polynesia to Brazil. Emerg Infect Dis 2015;21:1887.

120. J Zhang, Jin X, Zhu Z, et al. Early detection of Zika virus infection among travellers from areas of ongoing transmission in China. J Travel Med 2016;23.

121. N Rafiei, Hajkowicz K, Redmond A, Taylor C. First report of Zika virus infection in a returned traveller from the Solomon Islands. Med J Aust 2016;204:186-e1.

122. AT Pyke, Moore PR, Hall-Mendelin S, et al. Isolation of Zika Virus Imported from Tonga into Australia. PLoS Curr 2016;8.

123. EB Hayes. Zika virus outside Africa. Emerg Infect Dis 2009;15:1347-50.

124. Update on Zika virus transmission in the Pacific islands, 2007 to February 2016 and failure of acute flaccid paralysis surveillance to signal Zika emergence in this setting. Bull World Health Organ E-pub: 19 Feb 2016. doi: <http://dx.doi.org/10.2471/BLT.16.171892>, 2016. (Accessed 9th Nov, 2016, at <http://www.who.int/bulletin/online_first/16-171892.pdf.>)

125. International SOS (iSOS): Zika Virus. (Accessed 20 Nov, 2016, at https://[www.internationalsos.com/topics/zika-virus.](http://www.internationalsos.com/topics/zika-virus.))
